# Kv1.3 contains an alternative C-terminal ER exit motif and is recruited into COPII vesicles by Sec24a

**DOI:** 10.1186/s12858-015-0045-6

**Published:** 2015-07-10

**Authors:** John M. Spear, Dolly Al Koborssy, Austin B. Schwartz, Adam J. Johnson, Anjon Audhya, Debra A. Fadool, Scott M. Stagg

**Affiliations:** Institute of Molecular Biophysics, Florida State University, 91 Chieftan Way, Tallahassee, FL 32306 USA; Program in Neuroscience, Florida State University, 1107 West Call Street, Tallahassee, FL 32306 USA; Biomolecular Chemistry, University of Wisconsin-Madison, 440 Henry Mall, Madison, WI 53706 USA; Department of Biological Science, Florida State University, 319 Stadium Drive, Tallahassee, FL 32306 USA; Department of Chemistry and Biochemistry, Florida State University, 91 Chieftan Way, Tallahassee, FL 32306 USA

**Keywords:** Early secretory pathway, Anterograde intracellular trafficking, COPII, Endoplasmic reticulum, Voltage-gated potassium channel, Di-acidic motif, siRNA

## Abstract

**Background:**

Potassium channels play a fundamental role in resetting the resting membrane potential of excitable cells. Determining the intracellular trafficking and localization mechanisms of potassium channels provides a platform to fully characterize their maturation and functionality. Previous investigations have discovered residues or motifs that exist in their primary structure, which directly promote anterograde trafficking of nascent potassium channels. Recently, a non-conical di-acidic motif (E483/484) has been discovered in the C-terminus of the mammalian homologue of the *Shaker* voltage-gated potassium channel subfamily member 3 (Kv1.3), and was shown to disrupt the anterograde trafficking of Kv1.3.

**Results:**

We have further investigated the intracellular trafficking requirements of Kv1.3 both *in vivo* and *in vitro*. First, three alternative C-terminal acidic residues, E443, E445, E447 were probed for their involvement within the early secretory pathway of Kv1.3. Single point (E443A, E445A, and E447A) and double point (E443A-E445A, E445A-E447A) mutations exhibited no significant changes in their endoplasmic reticulum (ER) retention. The triple point mutant E443A-E445A-E447A displayed a modest ER retention while deletion of the C-terminus showed dramatic ER retention. Second, we demonstrate *in vivo* the requirement for the Sec24a isoform to confer anterograde trafficking using a siRNA knockdown assay. Third, we show *in vitro* the association of recombinantly expressed Kv1.3 and Sec24a proteins.

**Conclusion:**

These results expand upon previous studies aimed at deciphering the Kv1.3 secretory trafficking mechanisms and further show *in vitro* evidence of the association between Kv1.3 and the COPII cargo adaptor subunit isoform Sec24a.

**Electronic supplementary material:**

The online version of this article (doi:10.1186/s12858-015-0045-6) contains supplementary material, which is available to authorized users.

## Background

Intracellular protein secretion is an essential and complex process found in all eukaryotic organisms [1]. Approximately one-third of all eukaryotic proteins undergo transport from the endoplasmic reticulum (ER) to the ER-Golgi intermediate complex (ERGIC). This pathway is termed the anterograde early secretory pathway and is facilitated by the coat protein complex II (COPII) proteins [2]. The COPII secretory machinery is composed of five cytosolic proteins; Sar1, Sec23, Sec24, Sec13, and Sec31, which assemble in a processive and stepwise fashion (for review see [3, 4]). Many protein types travel through this pathway including a large class of integral membrane proteins known as the voltage-gated potassium channels (Kv channels) [5]. Kv channels are molecular machines that are selective for and conduct potassium ions across cellular membranes in response to changes in membrane potential [6]. There are forty human Kv channel family members that account for more than half of the seventy-eight potassium channel proteins identified in the human genome [7, 8].

The spatial and temporal distribution of Kv channels is a critical determinant of cell excitability and therefore these proteins remain a concentrated target of pharmacological drug studies to alleviate neuroinflammatory and autoimmune diseases [9–16]. Moreover, recent investigations have identified their peripheral roles in the regulation of metabolism, body weight, and management of type II diabetes [17–24]. Although, it has been found that Kv channels contain numerous signaling domains, which are primarily located on the cytoplasmic N- and C-termini, the mechanism(s) by which Kv channels reach their functional locations have not been completely characterized [25–27]. It has been shown that the N-terminus contains the T1 tetramerization domain, which promotes the formation of properly assembled channels [28, 29], while the C-terminus is recognized to harbor the ER export motif that facilitates efficient processing and localization of Kv channels to the plasma membrane [5, 30–33].

Several sequences have been shown to be associated with ER export of potassium channels. In the Kv1 family, a highly conserved C-terminal motif, HRETE has been shown to contribute to channel trafficking of Kv1.1, Kv1.2, and Kv1.4 [32] (Additional file 1, red box). A different motif, VxxSL, where x represents any amino acid followed by Ser then Leu, was discovered in Kv1.4 (Additional file 1, black box). This sequence was reported to have a significant role in that protein’s intracellular trafficking [34–36]. In Kv1.3, a non-canonical di-acidic ER export motif was discovered at positions E483-E484 [33] (Additional file 1, blue box). This motif is located further downstream from the previously reported HRETE motif (residues 441-445 of mouse Kv1.3) and was suggested to associate with Sec24d to promote the anterograde trafficking of Kv1.3 channels within the secretory pathway. Di-acidic exit motifs found in the C-termini of other potassium channels include VLSEVDETD in the inward rectifier (Kir) Kir1 subfamily, FCYENE in Kir2.1, ELETEEEE and NQDMEIGV in the Kir3.2A and Kir3.4 family members respectively, and EDE in the two-pore potassium channel (K_2p_) TASK-3 proteins [30, 37]. Interestingly, these potassium channel families share little sequence homology in their C-termini, yet their ER export mechanism(s) show a dependence on a di-acidic motif for efficient passage through the anterograde early secretory pathway.

The COPII cargo adaptor subunit Sec24 has four human isoforms (Sec24a-d) where different isoforms are recognized to bind to or associate with unique anterograde trafficking motifs to mediate ER export [38, 39]. Sec24 isoforms a and b have been shown to associate primarily with a di-acidic motif, D/ExD/E, where D/E are acidic residues, while Sec24 c and d recognize a series of hydrophobic residues [40–43]. Many of the ER exit motifs in potassium channel families contain di-acidic motifs, and mutations in these regions have been shown to disrupt potassium channel cell surface localization [5, 30, 32, 33, 37]. A putative canonical di-acidic motif is present on the cytoplasmic C-terminus of mouse Kv1.3 between residues 443-447 (ETEGE) that is partially contained in and extends beyond the HRETE sequence previously shown to contribute to the trafficking of Kv1.1, Kv1.2, and Kv1.4 (Additional file 1). Here, we show that this stretch of acidic residues has a modest yet measureable effect on the anterograde trafficking of Kv1.3 channels. Furthermore, we show a disruption of Kv1.3 anterograde trafficking upon knockdown of Sec24a and demonstrate that Kv1.3 is a bona fide client of this cargo adaptor isoform using an *in vitro* membrane flotation assay.

## Results

### Fluorescent tag of Kv1.3 channel does not perturb biophysical properties

Although it has been previously examined in other expression systems, we confirmed that our N-terminal enhanced green fluorescent protein (eGFP) insertion did not alter Kv1.3 channel function. Kv1.3-eGFP biophysics were compared to wild type (wt) Kv1.3 using patch-clamp electrophysiology. Kv1.3-eGFP exhibited nearly identical biophysical characteristics as that of wtKv1.3. Addition of the N-terminal eGFP did not significantly alter the magnitude of the peak or sustained current, the current-voltage relationship, the voltage at half activation (V_1/2_), or the kinetics of inactivation or deactivation (Additional file 2). Overall, these results suggest that there are no deleterious alterations of the Kv1.3 channel properties when fused with the eGFP reporter.

### Identifying the role of the Kv1.3 C-terminus in the anterograde early secretory pathway

**Fig. 1:**
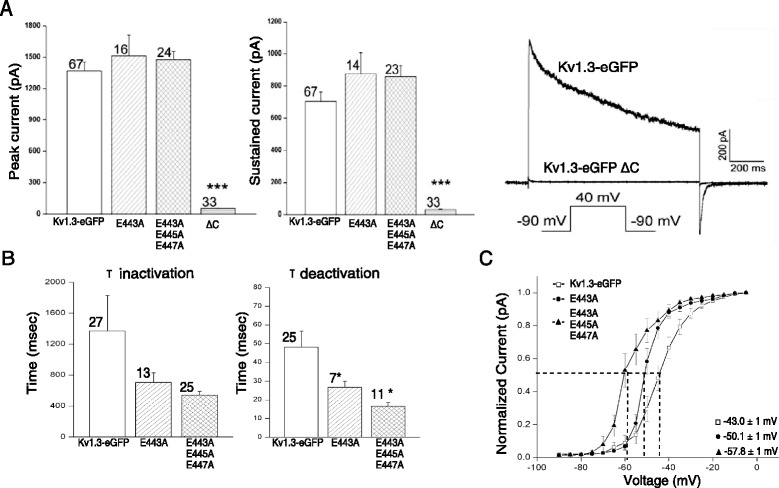
Biophysical properties of Kv1.3 channels following mutations of the acidic ER export motif. **a** Bar graph of the mean peak (left) or sustained (middle) current (± s.e.m.) for various voltage-clamped Kv1.3-eGFP or mutant channels as recorded in cell-attached patches using a single step depolarization of +40 mV (V_c_) from a holding potential (V_h_) of -80 mV. Representative current traces comparing Kv1.3-eGFP with that of Kv1.3-eGFP ∆C (right). **b** Same as in (A) but comparing inactivation (left) or deactivation (middle) kinetics of Kv1.3-eGFP. Significantly different by one-way ANOVA, Bonferoni’s post-hoc test, * = 0.001. **c** Line graph of the normalized tail currents is fit with a Boltzmann relation to calculate voltage at half-activation (V_1/2_). Significantly different V_1/2_ by one-way ANOVA, Bonferoni’s post-hoc test, *** = 0.0001, * = 0.001

**Fig. 2:**
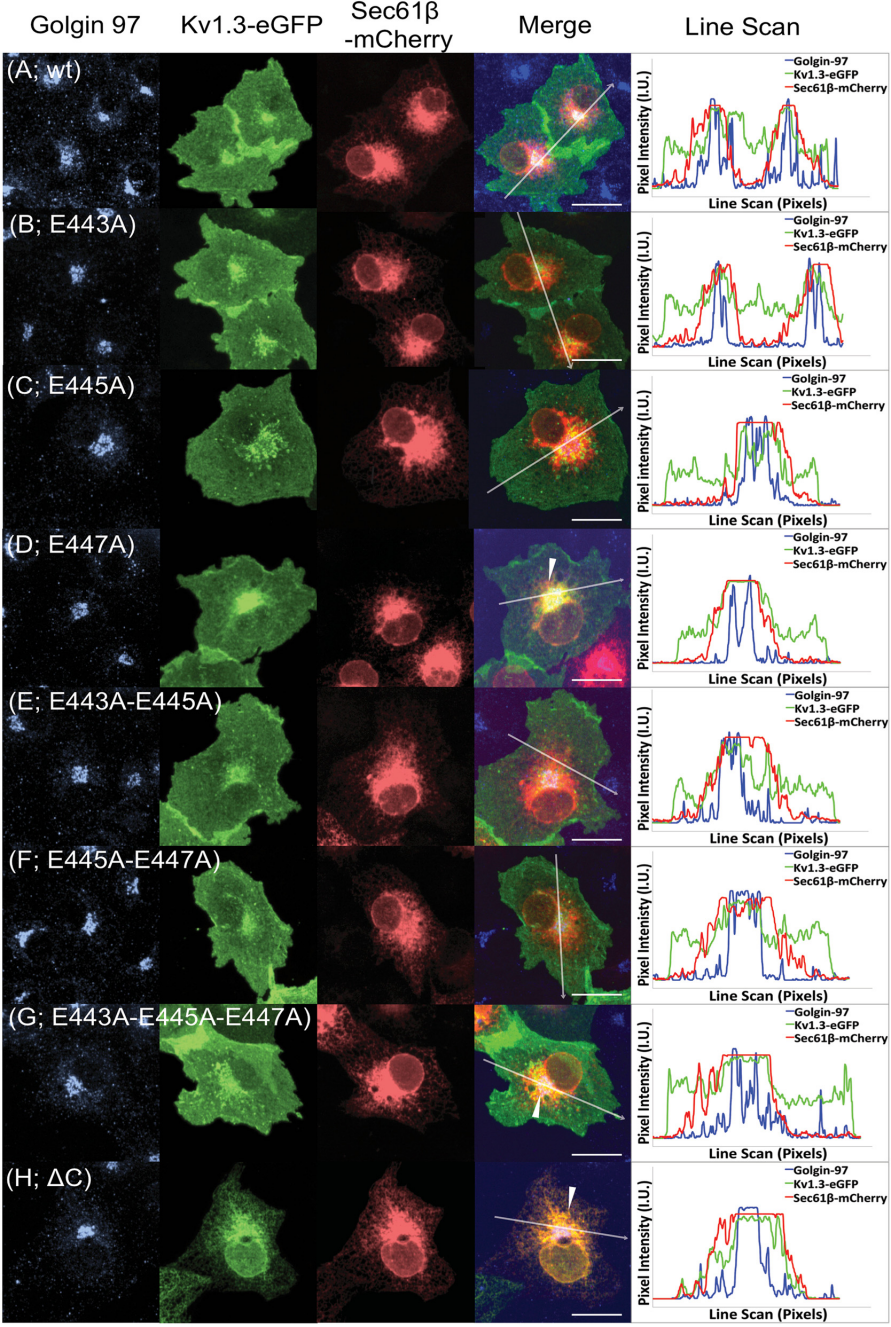
Localization of Kv1.3 channels after sequential mutation of the acidic ER export motif. The Kv1.3-eGFP and each of the mutated Kv1.3-eGFP proteins (see methods) were transiently expressed in BSC40 cells and the fluorescence was observed using confocal microscopy (**a**-**h**) in the presence of the membranous ER resident protein Sec61β and the Golgi resident protein Golgin97. The Kv1.3-eGFP co-localization with these organelle specific resident proteins and the resulting fluorescent trafficking profile (**a**) is similar in appearance with the Kv1.3-eGFP E443A (**b**), Kv1.3-eGFP E445A (**c**), Kv1.3-eGFP E443A-E445A (**e**), and Kv1.3-eGFP E445A-E447A (**f**) profiles indicating that there is no significant ER retention of any of these mutated proteins. A noticeable difference in the co-localization of Kv1.3-eGFP and Sec61β is observed in the Kv1.3-eGFP E447A (**d**), Kv1.3-eGFP E443A-E445A-E447A (**g**), and Kv1.3-eGFP ∆C (**h**) trafficking profiles where there is either a modest (**d** and **g**) or severe (**h**) retention within the ER network (**d**, **g**, and **h**; white arrows). Line scans of each fluorescent channel are shown. The white arrow in the merged image represents the placement and direction of the line scan. Scale bar = 10 μm

We first set out to determine the role of the Kv1.3-eGFP C-terminus in its anterograde early secretory trafficking mechanism. Upon deletion of the C-terminus (Kv1.3-eGFP ΔC), HEK 293 cells expressing the Kv1.3-eGFP ΔC channel showed a dramatic reduction in the outward current as compared to Kv1.3-eGFP as studied by patch-clamp electrophysiology (Fig. 1a). To better understand the consequences of deleting the C-terminus of Kv1.3-eGFP, we visualized the trafficking defect using confocal microscopy in BSC40 cells. Kv1.3-eGFP ΔC was highly retained in the ER tubular network as seen by its co-localization with the membranous ER resident protein Sec61β (Fig. 2h; white arrows). Thus, the C-terminus of Kv1.3 was a major determinant in promoting the ER export of Kv1.3 channels.

### C-terminal acidic motif ExExE mutations moderately alter Kv1.3 channel subcellular distribution

The C-terminus of Kv1.3 contains the conserved sequence HRET(D/E)xE (Additional file 1, red box) as a tandem di-acidic motif (ExExE), which we hypothesized could interact with Sec24 to facilitate ER export. Moreover, the HRETE motif had previously been shown in Kv1.1, Kv1.2, and Kv1.4 to contribute to trafficking of those proteins [32]. To examine the influence of the ExExE acidic motif in Kv1.3, we screened these acidic residues for their involvement to promote surface localization by patch-clamp electrophysiology after mutation using site-directed mutagenesis. The assumption was made that the degree of surface localization or channel density was proportional to current magnitude. There were no significant changes in the mean peak or sustained current magnitude of Kv1.3-eGFP E443A, or the Kv1.3-eGFP E443A-E445A-E447A mutants. The kinetics of inactivation for the E443A and E443A-E445A-E447A mutant channels demonstrated a strong trend to decrease the inactivation rate but did not reach statistical significance. The rate constant for the deactivation of the E443A and E443A-E445A-E447A channels was significantly less than that of the Kv1.3-eGFP channel (Fig. 1b). Finally, there was a significant increase in the voltage dependence for both the E443A and E443A-E445A-E447A mutant channels. The voltage at half-activation shifted to the left (Fig. 1c) 6 and 13 mV, respectively, representing a substantial change in movement of the channel at a more hyperpolarized state. While these changes in kinetics and voltage sensitivity would not be predicted to be involved in Kv1.3 trafficking, they represent changes in channel function attributed to manipulation of the acidic C-terminal motif where the Kv1.3 channel is driven to a more excitable state by reaching the activation threshold at more hyperpolarized potentials. It was not possible to explore these secondary biophysical changes for the Kv1.3-eGFP ∆C construct due to lack of resolvable current.

To further understand the ExExE acidic motif, we visualized the Kv1.3-eGFP localization using confocal microscopy. Single (Kv1.3-eGFP E443A and Kv1.3-eGFP E445A) and double point mutations (Kv1.3-eGFP E443A-E445A and Kv1.3-eGFP E445A-E447A) were found to be efficiently trafficked to the cell surface of BSC40 cells (Fig. 2a-c and e-f). Kv1.3-eGFP E447A and the triple point mutant Kv1.3-eGFP E443A-E445A-E447A, however, were moderately co-localized with the membranous ER resident protein Sec61β (Fig. 2d and g, respectively; white arrows). Line scans of each fluorescent channel further demonstrate the overlap of these two proteins (Fig. 2, right panel). These results demonstrate a moderate dependence on the acidic stretch of residues ExExE found within the C-terminus of Kv1.3.

### Kv1.3-eGFP ER retention on a population level

**Fig. 3:**
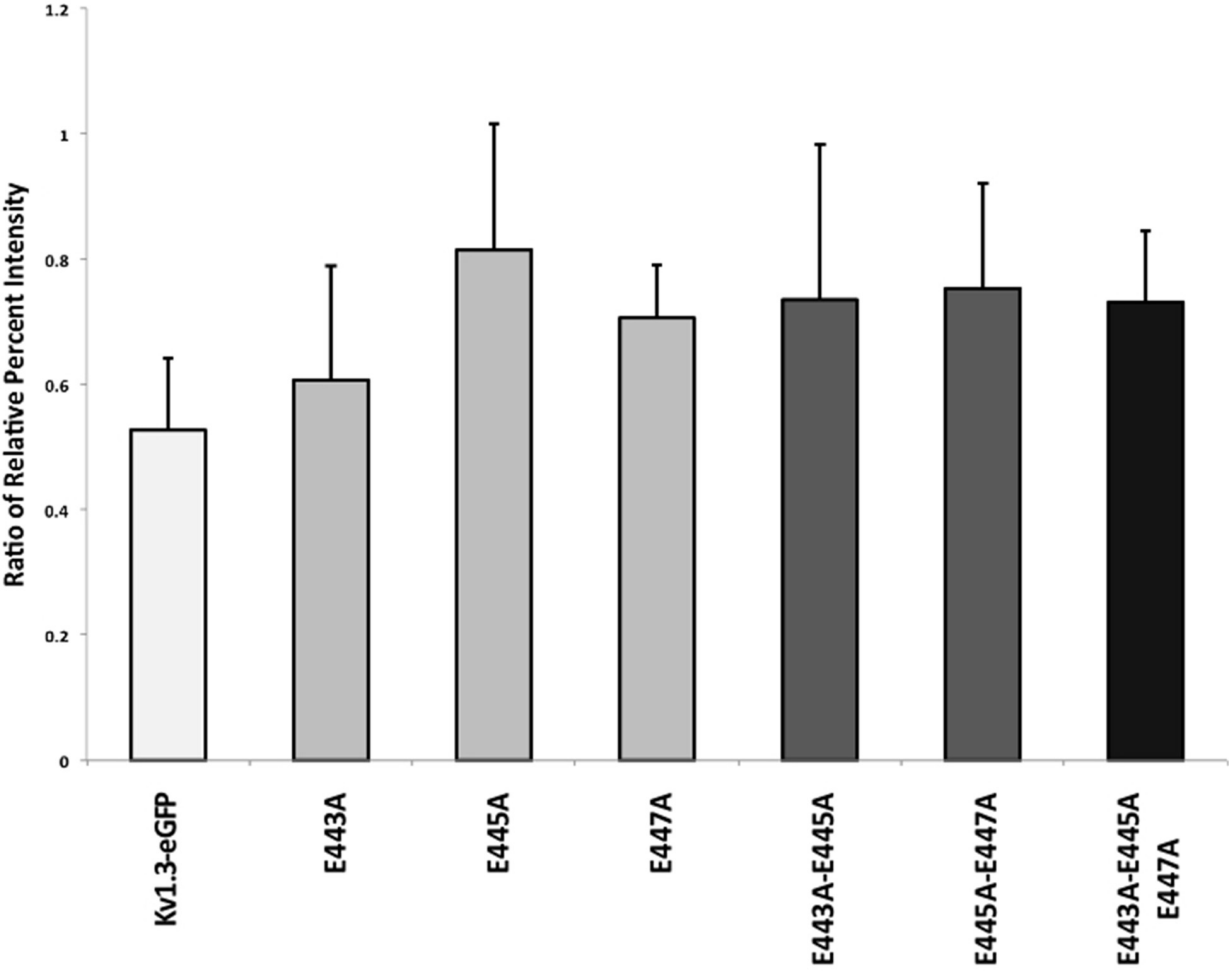
Retention of Kv1.3-eGFP in the ER upon sequential mutation of the acidic motif. Bar graph depicting the amount of Kv1.3-eGFP or mutant proteins retained in the ER. Ratio of relative percent intensity is equal to the amount of protein retained in the ER microsome fractions divided by the total amount of protein from whole cell homogenates. Resulting values were plotted as the mean ± standard error of the mean of three replicates (*n* = 3). The only statistically different mutant was the Kv1.3-eGFP E443A-E445A-E447A by one-way ANOVA, Bonferoni correction applied for Type-1 errors (p > 0.008)

Although our patch-clamp results suggested that the surface density was unchanged between Kv1.3-eGFP or mutant proteins, that measurement doesn’t account for the relative amounts of ER retention. To better understand the role of the acidic motif of Kv1.3 in ER trafficking, we compared the relative amounts of Kv1.3 in the ER over the total Kv1.3 expression for the different Kv1.3-eGFP mutants. The assays were conducted at 36 h post transfection across a population of HEK 293 cells as previous studies have shown high expression levels at that time point in that expression system [26]. ER retention of expressed Kv1.3-eGFP or mutant proteins was quantified by isolating ER microsomes from HEK 293 cells then using SDS-PAGE followed by Western blot densitometry analysis to determine the relative amounts of Kv1.3-eGFP protein retained in the ER microsomes. The ER microsome isolation was verified by probing for the luminal ER resident protein BiP, the luminal Golgi resident protein GCP60, the nuclear and perinuclear heat shock protein of 90 kDa (HSP90), and the cytosolic protein glyceraldehyde 3-phosphate dehydrogenase (GAPDH) (Additional file 3A). The relative amount of carryover from the GCP60, HSP90, and GAPDH proteins is low compared to the amount of BiP present in the final pellet indicating that our microsomes are representative of the contents of the ER. ER microsome isolations from cells expressing the Kv1.3-eGFP proteins were also probed for BiP (Additional file 3B) indicating that the ER microsomes from the cells expressing Kv1.3-eGFP or the respective mutant proteins remain intact through the isolation procedure. Western blots probing for Kv1.3-eGFP protein (Additional file 3C) were further subjected to computer-assisted densitometry where the amount of total protein found in homogenized HEK 293 cells was compared to the amount of Kv1.3-eGFP protein retained within the ER microsome enriched fraction (Fig. 3). The amount of Kv1.3-eGFP protein remaining in the ER was less (~30 %) when compared to both the Kv1.3-eGFP E445A and the Kv1.3-eGFP E443A-E445A-E447A mutant (*n* = 3; *p* = 0.008). This demonstrated that more of the Kv1.3-eGFP protein was exported from the ER than the Kv1.3-eGFP E443A-E445A-E447A mutant protein. Pairwise statistical comparisons between Kv1.3-eGFP and all other mutants determined that only the triple point mutant Kv1.3-eGFP E443A-E445A-E447A had a statistically significant amount of protein retained in the ER. The retention assay could not be used to determine the amount of Kv1.3-eGFP ΔC protein retained within the ER because the epitope used to generate the Kv1.3 channel antibody, FSU 120, was located on the C-terminus [44]. These results indicate that the acidic motif found within the C-terminus of Kv1.3 was not entirely dispensable, but was also not an exclusive requirement for Kv1.3 trafficking within the early anterograde secretory pathway.

### ER retention of Kv1.3-eGFP after siRNA knockdown of Sec24

**Fig. 4:**
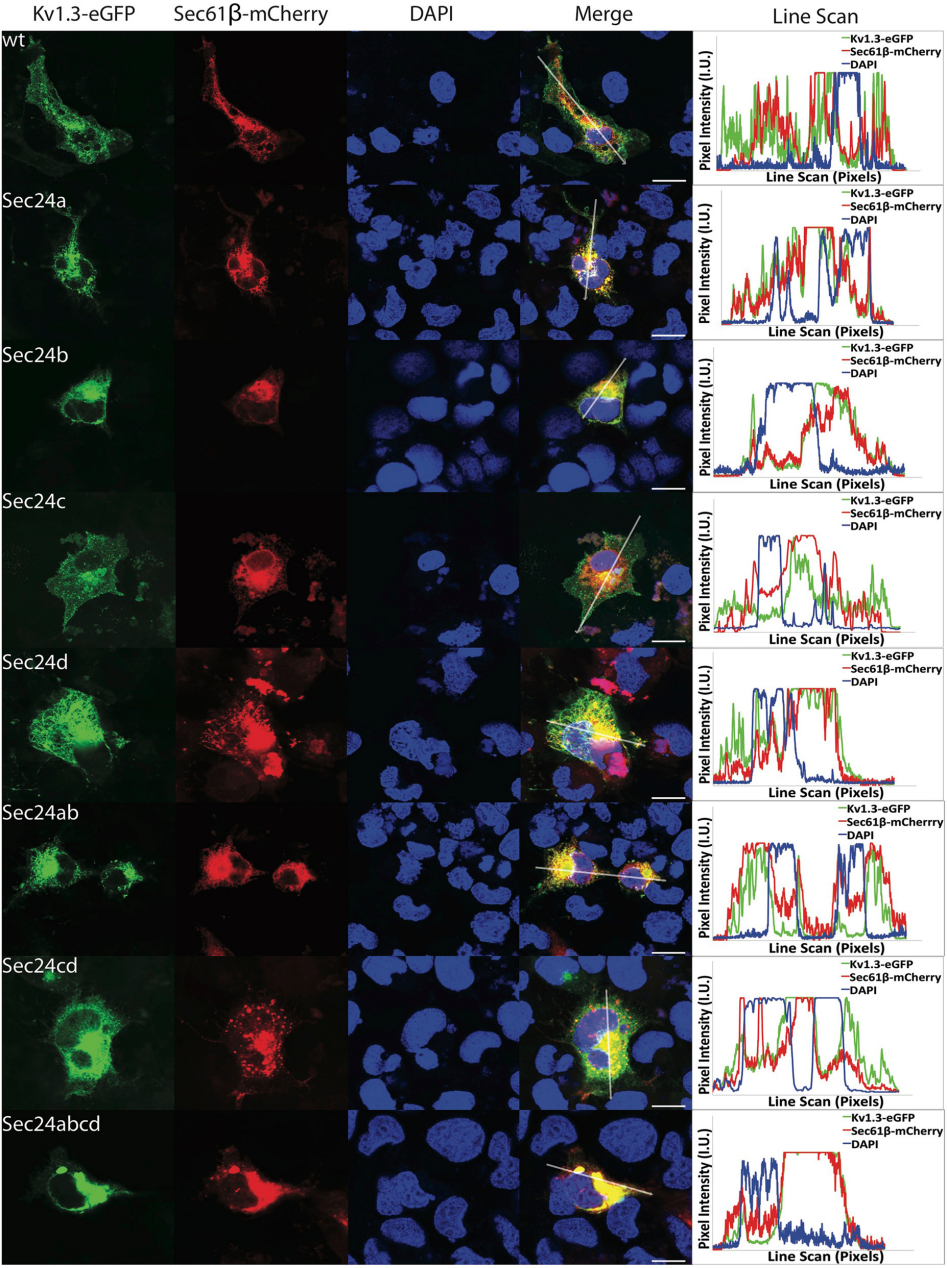
Kv1.3-eGFP trafficking after siRNA mediated knockdown of Sec24. Kv1.3-eGFP trafficking was examined after the knockdown of Sec24 isoforms (as indicated) in the presences of the membranous ER resident protein Sec61β tagged with the mCherry fluorophore (Sec61β-mCherry). Cellular nuclei were stained with DAPI. The wild-type (wt) trafficking profile is similar to the trafficking profile of Sec24c and Sec24cd knockdown conditions. An altered trafficking profile is seen in Sec24a, Sec24b, Sec24ab, and Sec24abcd conditions. Interestingly, there is also an altered trafficking profile in the Sec24d condition, but the Kv1.3-eGFP signal does not overlap well with the Sec61β protein. Line scans of each fluorescent channel are shown. The white arrow in the merged image represents the placement and direction of the line scan. Scale bar = 5 μm

In order to determine which Sec24 cargo adaptor subunit isoform was associated with Kv1.3 within a COPII vesicle, we used an RNA interference (siRNA) assay to specifically target each Sec24 isoform. The knockdown of each Sec24 isoform was established by probing silenced COS-1 cells via Western blot using isoform specific antibodies (Additional file 4). In the conditions used, all isoforms were knocked down. To better understand the Kv1.3-eGFP trafficking patterns upon Sec24 knockdown, we first co-transfected silenced COS-1 cells with cDNAs encoding Kv1.3-eGFP and the vesicular stomatitis virus glycoprotein (VSVG) tagged with a cerulean reporter (VSVG-Cerulean), the latter of which has been extensively characterized in the early secretory pathway [40, 45–48]. VSVG is a known client of Sec24a and Sec24b. Kv1.3-eGFP trafficking also showed a dependence on the Sec24a and Sec24b isoforms as observed via confocal microscopy. There were no dramatic changes in the intracellular trafficking profiles upon Sec24c, Sec24d, and Sec24cd knockdown (Additional file 5). The intracellular trafficking profile of the VSVG-cerulean protein exhibited similar features as the Kv1.3-eGFP trafficking profile as these proteins almost completely co-localized with each other in all experimental conditions. This observation is further demonstrated by their respective line scan profiles (Additional file 5, right panel). To further explore the Kv1.3-eGFP trafficking disruption caused by knockdown of Sec24, silenced COS-1 cells were co-transfected with cDNAs encoding Kv1.3-eGFP and the membranous ER resident protein Sec61β-mCherry cDNAs (Fig. 4). Kv1.3-eGFP co-localized with Sec61β in the Sec24a, Sec24b, Sec24a-b, and Sec24a-d, but not in the Sec24c, Sec24d, or Sec24cd knock down conditions. The intracellular fluorescent trafficking profile for Kv1.3-eGFP was similar to that of the Kv1.3-eGFP ∆C mutant upon double knockdown of Sec24a-b and also Sec24a-d (compare Fig. 4 with Fig. 2h) showing a strong retention of Kv1.3-eGFP within the endomembrane tubular network. Line scans of each fluorescent channel further demonstrate the overlap of these two proteins (Fig. 4, right panel). These results indicated that the early anterograde secretion of Kv1.3-eGFP protein from the ER relied upon the Sec24a and Sec24b isoforms.

### Kv1.3-Sec24a binding assessment by membrane floatation assay

**Fig. 5:**
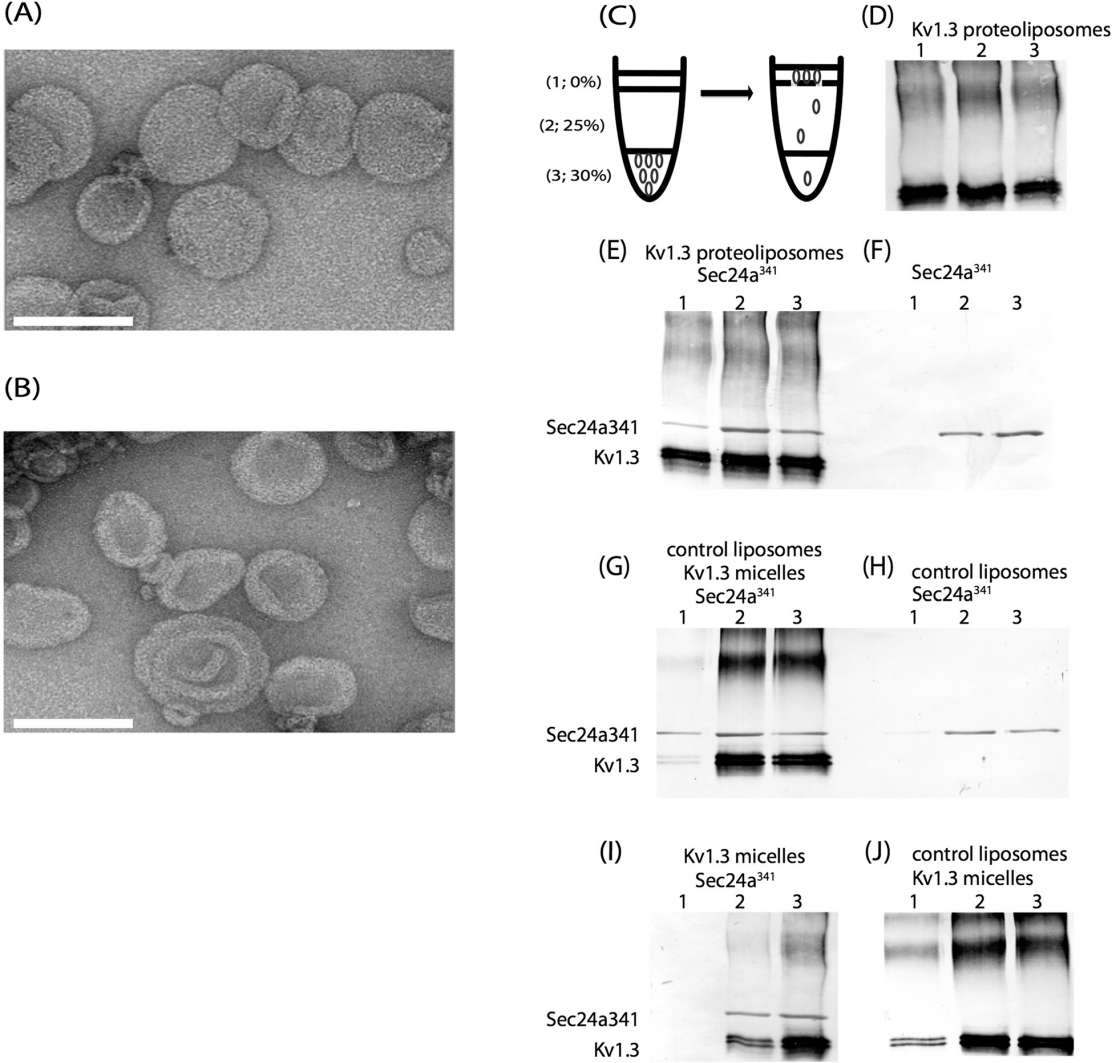
*In vitro* Kv1.3-Sec24a membrane floatation assay. Membrane floatation assay used to test for the association between Kv1.3 and Sec24a^341^. (**a**) Kv1.3 proteins reconstituted into synthetic lipid vesicles (proteoliposomes) and (**b**) control lipid vesicles (liposomes). (**c**) Schematic of the floatation assay. Proteoliposomes, drawn as small black circles, migrate through the three-step sucrose gradient (0 %, 25 %, and 30 % w/v sucrose; top (1), middle (2) and bottom (3), respectively) after incubation and centrifugation. (**d**) Kv1.3 proteoliposomes were found in the top fraction after centrifugation. (**e**) When Kv1.3 proteoliposomes (~65 kDa as a monomer) were incubated with Sec24a^341^ (~80 kDa), both Kv1.3 and Sec24a^341^ were detected in the top fraction. (**f**) Sec24a^341^ alone was not detected in the top fraction. (**g**) When Kv1.3 proteins in detergent micelles were mixed with Sec24a^341^ in the presence of control liposomes, both Kv1.3 and Sec24a^341^ were found in the top fraction. (**h**) Sec24a^341^ incubated with control liposomes was not found in the top fraction. (**i**) Kv1.3 micelles and Sec24a^341^ do not float in the absence of membranes. (**j**) Kv1.3 was detected in the top fraction when Kv1.3 in detergent micelles were incubated with control liposomes. Scale bar = 100 nm

To further examine the association between Kv1.3 and Sec24a, we used a membrane flotation assay to demonstrate that Kv1.3 is a client of Sec24a. Kv1.3 tetramers were characterized by native gel electrophoresis and negative stain electron microscopy (EM) (Additional file 6B and D-E). These analyses showed that solubilized and purified protein remained tetrameric. Interestingly, purified Kv1.3 proteins exchanged into the amphipol A8-35 appeared as a double band on 8 % SDS-PAGE. After addition of calf intestinal phosphatase (CIP), the double band collapsed to a single band (Additional file 6C). These results agree with previous studies that showed how Kv1.3 could exist at multiple molecular weights depending on its state of phosphorylation [49]. Solubilized but non-dephosphorylated tetramers were then reconstituted into lipid bilayers, producing proteoliposomes (Fig. 5a). Proteoliposomes are less dense than protein alone and floated to the top of a 25 % sucrose layer after centrifugation (Fig. 5c and d). When Kv1.3 proteoliposomes were incubated with Sec24a^341^ (Sec24a truncated to position 341) proteins, both Kv1.3 and Sec24a^341^ were detected in the top fraction (Fig. 5e) while Sec24a^341^ proteins in the absence of Kv1.3 and or control liposomes (Fig. 5b) were not detected in the top fraction but were concentrated in the middle and bottom fractions (Fig. 5f). Interestingly, when Kv1.3 in detergent micelles was mixed with Sec24a^341^ proteins in the presence of control liposomes, Kv1.3 and Sec24a^341^ proteins are found in the top fraction (Fig. 5g). This is attributed to the fact that Kv1.3 in detergent micelles can reconstitute into membranes and therefore recruit Sec24a^341^. This was verified by incubating either solubilized Kv1.3 proteins or Sec24a^341^ proteins in the presence of control liposomes. In these cases, Kv1.3 was found in the top fraction (Fig. 5j) while Sec24a^341^ proteins were not (Fig. 5h). Neither solubilized Kv1.3 nor Sec24a^341^ float in the absence of lipids (Fig. 5i). These results further demonstrate that Kv1.3 is a *bona fide* client of Sec24a.

## Discussion

Our results have shown that there is a modest yet measurable dependence on the acidic motif (ExExE) found within Kv1.3 to promote anterograde transport through the COPII mediated early secretory pathway. Our data indicate that the canonical di-acidic motif under investigation is an alternative moderator of ER exiting, and likely functions together with the downstream signal E483-E484 found by Martínez-Mármol *et al* [33] to promote robust and efficient trafficking of Kv1.3 proteins. Adding to the complexity of Kv channel trafficking dynamics is the fact that Kv channels are known to form heteromeric assemblies. A heteromeric Kv1.3 channel containing one or more different alpha subunit(s) that possesses an intact ER export motif(s) would be able bypass the Kv1.3 trafficking defect and enter the anterograde secretory pathway [50]. Furthermore, Kv channels have several interacting partners, such as the Kv beta subunit and the 14-3-3 proteins [51–54], each of which are capable of rescuing a defect within the secretory pathway. These interactions likely all work together to facilitate robust and efficient Kv1.3 trafficking in the cell.

We have shown that deletion of the C-terminus of Kv1.3 produces a dramatic ER retention. This finding is in good agreement with other recent investigations that probed the trafficking defect produced after removal of the Kv1.3 C-terminus [33]. Our patch clamp analysis demonstrated that there is little to no current at the plasma membrane of HEK 293 cells expressing the Kv1.3-eGFP ΔC protein. We were not able to distinguish whether lack of current indicates that the Kv1.3-eGFP ΔC protein was retained due to improper folding within the ER. Previous structural and biophysical characterization of Kv channels has found that the C-terminus of *Shaker* channels are intrinsically disordered [55]. It is likely that deleting the disordered C-terminus of Kv1.3 would have a minimal effect, or none at all, on the tertiary and quaternary structure of Kv1.3. Taken together, we judge that removal of the Kv1.3 C-terminus probably impacts less upon protein folding and is more likely a functional loss of the ER export motif resulting in retention of the channel within the ER.

VSVG has been well documented to contain a di-acidic exit motif and to interact with the Sec24a and Sec24b isoforms [39, 40]. By using VSVG-cerulean as a trafficking control, we showed that the intracellular Kv1.3 trafficking profile is similar to that of VSVG in the presence and absence of siRNAs targeting individual Sec24 isoforms. When the Kv1.3-eGFP trafficking profile is observed together with VSVG-cerulean, there is a strong overlap between the two cargo proteins. When either or both Sec24a and Sec24b are knocked down, both Kv1.3 and VSVG are strongly retained. This demonstrates a strong dependence of Kv1.3 on Sec24a or Sec24b. Furthermore, we have shown that Sec24a^341^ directly binds membrane reconstituted or detergent solubilized Kv1.3 proteins using a membrane floatation assay. This *in vitro* interaction supports the *in vivo* data that suggested Kv1.3 is a client of Sec24a.

We have investigated the requirement for Kv1.3 channels to exit the ER by an acidic motif dependent mechanism. Even though the atomic architectures of Kv channels are starting to be elucidated, ambiguities still exist in how these proteins are processed and eventually targeted to their functional domains. Li *et al* [34] were the first to propose the VxxSL ER exit motif for Kv1 channels. This motif, however, is not conserved in the Kv1.3 channel. Zhu *et al* [32] showed that trafficking of Kv1.1, Kv1.2, and Kv1.4 channels depend on a conserved C-terminal set of residues including an HRETE motif, which overlaps with the residues we tested in Kv1.3. Moreover, the HRETE motif was not completely targeted by Martínez-Mármol *et al* [33], but a notable trafficking defect was observed when the E445A (in mouse Kv1.3, E442 in rat Kv1.3) residue was mutated. In that study, Martínez-Mármol *et al* [33] showed that trafficking of Kv1.3 critically depends on the E483-E484 acidic residues, and demonstrated an *in vivo* interaction with Sec24d [33]. Those authors make note that Sec24d co-localized with Kv1.3 only in the presence of elevated COPII coat protein Sar1b that functions to recruit the rest of the coat to the ER membrane. Furthermore, it has been well documented that Sec24d (and c) interacts with a stretch of hydrophobic residues while the Sec24a and b isoforms interact with a di-acidic motif [4, 43, 56–59]. It remains an open topic as to how the non-canonical di-acidic motif of Kv1.3 would associate with the Sec24d isoform. Martínez-Mármol *et al* [33] used computational modeling to model the structure of the Kv1.3 C-terminus in association with Sec24a *in lieu* of Sec24d. They postulated that Kv1.3 contacts Sec24a in their modeled structure and by extension Sec24d *in vivo* by forming two salt bridges between E483-E484 of the homology modeled Kv1.3 C-terminus (there is no structural information currently available for the C-terminus of Kv1.3) and R750, R752, and Y437 of Sec24 (these residues are conserved across all Sec24 isoforms (Additional file 7)). Though an explicit interaction between Kv1.3 and Sec24d cannot be ruled out, it remains unclear as to how the exclusive nature of this reported selectivity would be preserved if the E483-E484 residues could potentially bind any Sec24 isoform. Our Sec24 knockdown experiments, however, show that Kv1.3 exhibits a trafficking dependence on the Sec24a and Sec24b isoforms, which satisfies the current paradigm of how di-acidic motifs interact with the COPII cargo recruitment subunit isoforms.

## Conclusion

In conclusion, the trafficking mechanism(s) by which Kv1.3 exits the ER and travels through the anterograde early secretory pathway was explored. It was found that Kv1.3 is partially retained within the ER after complete mutation of the ExExE motif that is located between residues 443-447 of mouse Kv1.3, and nearly completely retained upon truncation of the C-terminus. Furthermore, Kv1.3 was shown to associate directly with the COPII cargo adaptor subunit Sec24a *in vitro* using a membrane flotation assay. Moreover, these results agree with previous studies aimed at unraveling the mechanism(s) by which Kv1.3 exits the ER and travels through the anterograde early secretory pathway [33]. Finally, these results could potentially aid in the development of pharmaceuticals directed towards autoimmune disease associated with the mis-targeting of Kv1 channels *in vivo* [60].

## Methods

### Cell culture

Human embryonic kidney-293 (HEK 293) cells were cultured as previous reported [25]. Briefly, HEK 293 cells were maintained in minimum essential medium (MEM) (Gibco), 2 % w/v penicillin/streptomycin (Sigma), 10 % fetal bovine serum (FBS) v/v (Gibco), and incubated at 37 °C with 5 % CO_2_. Cells were grown to 80 % confluency on poly-L-lysine treated Corning dishes (Fisher Scientific) with fresh media exchanges every 48 h. African green monkey kidney fibroblast-like (COS-1) cells were cultured in a nearly identical way to the HEK 293 cells with the following changes: COS-1 cells were maintained in MEM (Gibco), 2 % w/v penicillin/streptomycin (Sigma), 10 % FBS v/v (Gibco) and incubated at 37 °C with 5 % CO_2_. Cells were grown to 50 % confluency on poly-L-lysine treated Corning dishes (Fisher Scientific) with fresh media exchanges every 48 h.

### Construction of Kv1.3 channel mutants

Kv1.3 channels were expressed transiently in HEK 293 and COS-1 as previously described [25]. pCDM8 was a kind gift from Dr. Brian Seed (Harvard University) [61]. cDNA encoding human CD8 was amplified from pCDM8 and subcloned into the pcDNA_3_ vector as previously reported [62]. The eGFP reporter was fused to the N-terminus of Kv1.3 immediately after the start codon of Kv1.3 (Kv1.3-eGFP) at the Florida State University Molecular Cloning Facility as previously described [63]. Kv1.3 constructs containing the single point mutations (Kv1.3-eGFP E443A, Kv1.3-eGFP E445A, Kv1.3-eGFP E447A), double point mutations (Kv1.3-eGFP E443A-E445A, Kv1.3-eGFP E445A-E447A), triple point mutation (Kv1.3-eGFP E443A-E445A-E447) and the Kv1.3-eGFP C-terminal truncated protein (Kv1.3-eGFP ∆C) mutant were generated using the QuickChange® Mutagenesis kit (Stratagene) according to manufacturer’s specifications. The Kv1.3-eGFP ∆C cDNA has a stop codon at position 427 of Kv1.3 (Kv1.3-P427Stop). All constructs were sequenced to verify the mutation(s) and detect PCR errors.

### cDNA transient transfection

cDNA was introduced into HEK 293 and COS-1 cells with a LipofectAMINE reagent (Invitrogen) five to seven days after passage as previously described [25]. Briefly, HEK 293 and COS-1 cells were transfected for 4 h with 1.0 μg of each cDNA construct plus 4 μl of LipofectAMINE per 35 mm dish for electrophysiology and fluorescent microscopy. For Western blot analysis in HEK 293 and COS-1 cells, 6 μg of each cDNA construct plus 14 μl of LipofectAMINE per 60 mm dish was used. HEK 293 and COS-1 cells were either harvested for biochemical analysis, confocal microscopy, or used for electrophysiological recordings 36 h post-transfection, as previous studies have shown high surface expression levels at that time point [26]. For all transient transfections the lipofectamine and appropriate cDNAs were complexed for 20 min at room temperature.

### Patch-clamp electrophysiology

Thirty six hours post-transfection, HEK 293 cells were rinsed with bath solution (150 mM KCl, 10 mM HEPES, 1 mM EGTA, and 0.5 mM MgCl_2_, pH 7.4) and incubated with anti-hCD8 beads (Dynabeads®; Invitrogen) for 2 min to mark transfected cells [61, 62]. Co-expression with CD8 allowed visualization of cells taking up Kv1.3 cDNA by marking transfected cells with a red polypropylene-antibody-linked bead. Hoffman modulation contrast optics was used to visualize transfected HEK 293 cells at 40x magnification using an Axiovert 135 microscope (Zeiss). Transfected HEK 293 cells were rinsed two times with bath solution before beginning a recording session to remove any unbound beads. Patch pipettes were fabricated from #M15/10 borosilicate glass (Jencons) and were fire polished to approximately 1 μm (bubble number 5.0) [64]. Pipette resistances between 8 and 14 MΩ were used to produce high-resistance giga seals by applying a small amount of suction to the pipette lumen in contact with the target cell. HEK 293 cells were voltage-clamped at a holding potential (V_h_) of -90 mV and patches were generally depolarized to a command voltage (V_c_) of +40 mV for pulse durations of 1000 milliseconds. Pulses were typically delivered at intervals of 60 s to prevent cumulative inactivation of the Kv1.3 channel [65]. Macroscopic currents were recorded in the cell-attached configuration using an AxoPatch200B integrating patch-clamp amplifier (Molecular Devices). The analog output was filtered between 2-5 kHz and digitally sampled every 0.5 - 4 milliseconds. Data acquisition was carried out using pClamp 10 software (Molecular Devices). Data were subsequently analyzed using software from Microcal Origin (Northampton) and QuattroPro (Borland International). The inactivation of the macroscopic current (τ_inact_) was fitted to the sum of two exponentials (y = y_0_ + A_1_exp(x/t_1_) + A_2_exp(x/t_2_)) by minimizing the sums of squares, where y_0_ is the Y offset, t_1_ and t_2_ are the inactivation time constants, x is the time, and A_1_ and A_2_ are the amplitudes. The two inactivation time constants were mathematically combined by multiplying each by its weight (A) and summing. The deactivation of the macroscopic current (τ_deact_) was fitted similarly, but to only a single exponential (y = y_0_ + A_1_ exp(x/t_1_)). The voltage-dependent activation of Kv1.3 was determined by fitting current-conductance relationships with a Boltzmann expression. The Boltzmann equation that was used for fitting was: y = [(A_1_-A_2_)/(1 + exp(X -X_o_)/dx) + A_2_] [44, 66].

### Kv1.3-eGFP and C-terminal mutant localization studies

BSC40 cells at 50 % confluency were co-transfected with cDNAs encoding the membranous ER resident protein Sec61β fused with the mCherry reporter and various mutants of Kv1.3-eGFP using Fugene HD (Promega). Cells were maintained in Dulbecco’s Modified Eagle Medium (DMEM) supplemented with 10 % FBS, 1x penicillin and streptomycin, and L-glutamine at 37 °C in the presence of 5 % CO_2_. 24 h post-transfection, cells were fixed for 15 min at 37 °C in 4 % paraformaldehyde v/v, washed three times with Tris buffered saline (TBS) (50 mM Tris, 150 mM NaCl, pH 7.6) and permeabilized with 0.5 % v/v Triton X-100 for 10 min at room temperature. Immunolabelling was carried out using mouse anti-Golgin 97 primary antibodies (Life Technologies) and anti-mouse secondary antibodies conjugated to Alexa 647 (Jackson ImmunoResearch). Images were acquired using a swept-field confocal microscope (Nikon Ti-E) equipped with a Roper CoolSnap HQ2 charge-coupled device (CCD) camera using a Nikon 60x, 1.4 numerical aperture Planapo oil objective lens. Acquisition parameters were controlled by Nikon Elements software. Line scan profiles of the pixel intensity versus their location in the respective images for all fluorophores were generated using ImageJ. Line scans were drawn in order to maximize the amount of cellular content that could be measured in a given scan.

### ER microsome extraction

Intact HEK 293 ER microsome isolates were prepared as previously reported with the following modifications [67]. HEK 293 cells were cultured and transfected as stated above for biochemical analysis. Cell cultures were harvested by first washing two times with hypo-osmotic lysing buffer (2 mM KCl, 25 mM Tris, 5 μg/ml aprotinin (Sigma), 1 μg/ml pepstatin A (Sigma), 0.2 mM phenylmethanesulfonylfluoride (PMSF) (Sigma), and 1 μg/ml leupeptin (Sigma), pH 7.4). Once washed, cells were collected and resuspended in 5 mM HEPES pH 7.4 and stored at -20 °C. Frozen cell suspensions were thawed in a 18 °C water bath, centrifuged at 700 g for 7 min at 4 °C, resuspended in 5 mM HEPES pH 7.4, and homogenized with a Dounce homogenizer (7 strokes). Homogenates were cleared of any intact cells by centrifugation at 700 g for 7 min at 4 °C. 200 μg of the cleared cell homogenate samples were taken for Western blot analysis and immediately mixed with SDS-PAGE sample buffer (250 mM Tris, 10 % w/v SDS, 30 % v/v glycerol, 5 % v/v 2-mercaptoethanol, 0.02 % w/v bromophenol blue, pH 6.8) to a final concentration of 10 μg/μl and heated to 95 °C for 5 min. The remaining lysate was centrifuged at 18,000 g for 20 min at 4 °C. The supernatant was isolated and spun at 100,000 g for 45 min at 4 °C. The microsome enriched pellet was resuspended in SDS-PAGE sample buffer to a final concentration of 10 μg/μl and heated to 95 °C for 5 min. Protein concentrations were determined using a ND-1000 spectrophotometer (NanoDrop) and absorbance values at 280 nm were recorded. Control Western blots were performed to determine the efficiency of ER microsome isolation by probing for the luminal ER resident protein BiP (anti-BiP; Sigma), the luminal Golgi resident protein GCP60 (anti-GCP60; Origene), the nuclear and perinuclear heat shock protein of 90 kDa (HSP90) (anti-HSP90; Origene) and the cytosolic protein glyceraldehyde 3-phosphate dehydrogenase (GAPDH) (anti-GAPDH; Origene). Host-specific secondary antibodies conjugated with an alkaline phosphatase reporter (Sigma) were added after three washes in TBST for 1 h at room temperature. All Western blots were developed using the SIGMAFAST™ BCIP®/NBT tablets (Sigma). All antibodies were diluted in 5 % non-fat dry milk w/v in TBST to the following working dilutions: BiP (1:500), GCP60 (1:500), GAPDH (1:500), HSP90 (1:500) and all secondary antibodies (1:2500).

### Western blot and quantitative densitometry

Western blots were performed using standard biochemical techniques. 150-200 μg of total protein from the ER microsome and the corresponding homogenate samples were separated on independent 8 % SDS-PAGE gels. After visual separation of a pre-stained protein standard (BioRad), gels were electro-transferred to nitrocellulose (Pall Life Sciences) at 4 °C in Towbin transfer buffer (25 mM Tris-Base, 192 mM glycine, 10 % v/v methanol, pH 8.3). Membranes were first blocked with 5 % non-fat dry milk w/v in tween TBS (TBST) (0.05 % Tween-20 v/v; (Sigma)) for 1 h at room temperature. Blocked membranes were incubated overnight at 4 °C with Kv1.3 antisera, FSU120, generated against the intracellular C-terminal domain [44] and an anti-actin primary antibody (Millipore). Host-specific secondary antibodies conjugated with an alkaline phosphatase reporter (Sigma) were added after three washes in TBST for 1 h at room temperature. All antibodies were diluted in 5 % non-fat dry milk w/v in TBST to the following working dilutions: FSU120 (1:1000), anti-actin (1:2500),

Densitometry measurements were performed using the ImageJ software package as previously reported [68] with the following modifications. Western blots (*n* = 3 for both the homogenate and ER microsome enriched fraction) were digitized on an Epson Perfection 4490 Photo scanner and band intensities of Kv1.3-eGFP proteins located at ~100 kDa were recorded (the combined mass of a Kv1.3 monomer and eGFP). The band intensities recorded from homogenate and ER microsome enriched fractions expressing the channel or mutant proteins were rationalized by comparing the amount of Kv1.3-eGFP protein in the ER microsome enriched fraction to the total amount of Kv1.3-eGFP protein in the cleared homogenate sample. The anti-actin band was used as a visual loading marker to ensure all lanes had equal amounts of Kv1.3-eGFP proteins. All data are presented as mean ± standard error of the mean.

### siRNA silencing of Sec24

Knock down of all SEC24 isoforms (a-d) was performed using a siRNA silencing system (Invitrogen). COS-1 cells were transfected using predesigned stealth RNA duplex oligoribonucleotides (purchased as sets of three siRNAs per isoform from Invitrogen) and the appropriate controls as recommended by the manufacturer (Invitrogen). The supplier siRNA label codes were as follows: SEC24AHSS145804, SEC24AHSS145805, and SEC24AHSS145806 (for SEC24A); SEC24BHSS115967, SEC24BHSS115968, and SEC24BHSS173629 (for SEC24B); SEC24CHSS114388, SEC24CHSS114389, and SEC24CHSS114390 (for SEC24C); SEC24DHSS114919, SEC24DHSS114920, and SEC24DHSS190682 (for SEC24D). COS-1 cells were transfected using the RNAiMAX™ reagent (Invitrogen) according to manufacturer’s specifications to achieve a final siRNA concentration of 50 nM on cells. Negative controls (stealth RNAi negative control duplexes; Invitrogen) were included in each experiment as recommended by the manufacturer. 24 h following SEC24 silencing, COS-1 cells were tested via Western blot for Sec24 knockdown using primary antibodies specific to each Sec24 isoform (Cell Signaling Technologies; Sec24a: #9678S, Sec24b: #7427S, Sec24c: #8531S, Sec24d: 9610S). Western blots were performed using standard biochemical techniques as outlined above. All primary Sec24 antibody dilutions were at 1:333 and secondary antibody dilutions were at 1:2500 in 5 % non-fat dry milk w/v in TBST.

### Kv1.3-eGFP localization studies after Sec24 knock down

COS-1 cells were grown to 50 % confluency on poly-L-lysine coated glass cover slips (Thermo Scientific). Following siRNA treatment, COS-1 cells were further co-transfected with Kv1.3-eGFP and cDNAs encoding for either the vesicular stomatitis virus glycoprotein tagged with a cerulean fluorophore (VSVG-Cerulean) (Addgene; plasmid 11913) [45], or the membranous ER resident protein Sec61β tagged with the mCherry fluorophore (Sec61β-mCherry) (Addgene; 49155) [69] using the Lipofectamine™ 2000 reagent (Invitrogen) as described above. Following a 36 h transfection, cells were washed 3 times with phosphate buffered saline (PBS) solution (137 mM NaCl, 2.7 mM KCl, 10 mM Na_2_HPO_4_⋅2H_2_0, 2 mM KH_2_PO_4_, pH 7.4) and cellular nuclei were stained using 2-(4-amidinophenyl)-1H -indole-6-carboxamidine (DAPI) according to manufacturer’s recommendations (Invitrogen) then mounted onto clear microscope slides (Electron Microscopy Sciences) using the ProLong® Anti-Fade kit (Invitrogen) according to manufacturer’s specifications. Images were acquired from a Leica TCS SP2 AOBS confocal microscope equipped with the Chameleon TI:Sapphire multi-photon laser. All acquisition parameters were controlled within the Leica acquisition suite. Images were acquired using an Olympus 63X 1.4 numerical aperture oil immersion objective with a detector pinhole diameter set to 1 airy unit. Detector gain was kept under 800 using the Leica control panel to maintain equal exposure and detection of all emission spectra. 4 individual scans were acquired and averaged to create the final image. All Images were acquired in a 2048 × 2048 pixel array in LEI file format, and then were converted to a 16 bit TIFF file format using the Leica acquisition software. Line scans were drawn in the same fashion as described above.

### Statistical analyses

In electrophysiological studies, biophysical properties were compared across mutant Kv1.3-eGFP channels using an analysis of variance (ANOVA; one-way) with the channel as the independent variable and the biophysical property (i.e. peak current amplitude, voltage at half-activation, rate of inactivation, or rate of deactivation) as the dependent variable. Post-hoc multiple comparison tests were performed using a Bonferoni test. Current-voltage relationships were compared using a two-way ANOVA with the channel and voltage as independent variables and current as the dependent variable. Comparison of biophysical properties between wildtype and eGFP tagged Kv1.3 channels were made by Student’s t-test. For all electrophysiology experiments, the level of statistical significance was determined at the 95 % percent confidence level or greater and is noted by the p-value.

For Western blot densitometry studies, pairwise one-way ANOVAs were performed to determine if the mutated Kv1.3-eGFP protein was retained in the ER compared to the Kv1.3-eGFP protein. To adjust for the possibility of Type-1 errors, a Bonferoni correction was applied and yielded a new p-value of 0.008.

### Kv1.3 recombinant expression, purification, and reconstitution

Mouse Kv1.3 cDNA was cloned from the Kv1.3-pcDNA_3_ expression vector described above in the ‘Construction of Kv1.3 channel mutants’ section by enzymatic digestion at the unique Nco and Kpn restriction sites of the wild type Kv1.3-pcDNA_3_ vector. Kv1.3 cDNA was ligated in the pFastbac HT expression vector (Invitrogen) between the Nco and Kpn restriction sites. Cloning at these sites provided the addition of a TEV cleavable histidine tag (His_6_) downstream of the polyhedrin promoter using standard molecular techniques. The final recombinant expression vector was sequence verified to detect the presences of any PCR errors. The Kv1.3 pFastbac recombinant expression vector was transformed into competent DH10bac cells (Invitrogen) according to manufacturer’s recommendations. Two rounds of blue/white selection were performed and the Kv1.3 bacmid DNA was isolated using the Qiagen endotoxin free MaxiPrep kit (Qiagen). Baculovirus generation and amplification was performed by incubating 1 μg of Kv1.3 bacmid DNA that was complexed with 6 μl of the Cellfectin reagent (Invitrogen) and further transfected into Sf9 cells seeded at 1 x 10^5^ cells/mL in Graces modified media (Gibco) supplemented with 50 μg/ml gentamycin (Sigma) and 5 % FBS (Gibco). Sf9 cells were cultured at 27 °C with 5 % CO_2_ and constant shaking at 150 rpm. After 72 h the culture media was removed and used for further rounds of viral amplification as recommended by the manufacturer. Control infections were performed in parallel and did not receive bacmid DNA but only the Cellfectin reagent.

Kv1.3 proteins were expressed in Hi5 insect cells at a multiplicity of infection (MOI) of one after three consecutive rounds of virus amplification. Hi5 cells were cultured at 27 °C with 5 % CO_2_ and constant shaking at 150 rpm. Viral titers were greater than 1 x 10^9^ plaque forming units (pfu) pfu/mL. Hi5 cells were cultured in ESF-921 serum free media (Expression Systems) for 72 h post infection. Infected Hi5 cells were harvested by centrifugation at 700 g and pellets were stored at -80 °C until purification.

Kv1.3 proteins were purified as previously reported using a modified protocol [70]. Briefly, infected Hi5 cells were resuspended in Kv1.3 resuspension buffer (25 mM Tris, 150 mM NaCl, 150 mM KCl, one EDTA-free protease inhibitor cocktail (PIC) tablet (Roche) per 50 mL of buffer, pH 7.4). Resuspended Hi5 cells were lysed in a Microfluidizer (Microfluidic Corporation) and lysates were pre-cleared by centrifugation at 60,000 g. Lysate supernatants were cleared of any remaining membrane fragments by ultra centrifugation at 230,000 g. Both membrane-containing pellets were combined and resuspended in Kv1.3 solubilization buffer (resuspension buffer with 4 mM n-dodecyl phosphatidylcholine (Fos-12) (Affymetrix)). Kv1.3 proteins were solubilized overnight at room temperature on a rotating carousel. Insoluble material was removed by centrifugation at 60,000 g and the remaining supernatant was loaded onto a 25 ml column packed with Chelating Sepharose™ fast flow resin (GE Healthcare) charged with cobalt ions (Sigma) using an ÄKTA fast protein liquid chromatography (FPLC) machine (GE Healthcare). The column was equilibrated with 2 column volumes of purification buffer A (resuspension buffer containing 1 mM Fos-12) and Kv1.3 proteins were eluted with purification buffer B (purification buffer A with 500 mM imidazole (Sigma)). Eluted Kv1.3 proteins were concentrated to 5 mg/ml, loaded onto a Superose 6 10/300 GL column (GE Healthcare), and size excluded after the column was equilibrated with 2 column volumes of Kv1.3 purification buffer A using an ÄKTA FPLC. Eluted Kv1.3 proteins were analyzed for purity on a 12 % SDS-PAGE gel and fractions containing Kv1.3 protein were concentrated using a 100,000 kDa molecular weight cut off (MWCO) spin concentrator (Corning) to 1 mg/ml as determined by absorbance at 280 nm. Purified Kv1.3 proteins were stored at -80 °C until further use.

Kv1.3 proteins were reconstituted into membranes using a lipid composition of 95 %:5 % 1,2-Dioleoyl-sn-glycero-3-phosphocholine (DOPC): 1,2-dioleoyl-*sn-*glycero-3-phospho-L-serine (DOPS) w/w with a lipid to protein ratio of 1:1 mol:mol by detergent dialysis. Kv1.3 was dialyzed against detergent-free dialysis buffer (25 mM Tris, 150 mM NaCl, 150 mM KCl, 0.01 % NaN_3_ w/v, pH 7.5) in a 100,000 kDa MWCO slide-a-lyzer cassette (Thermo). Kv1.3 proteins were dialyzed for 1 week at room temperature in the presence of SM2 bio-beads (Bio-Rad) (1 g of washed and equilibrated bio-beads per liter of detergent-free dialysis buffer) with buffer changes every 24 h. Membrane reconstituted Kv1.3 proteins (termed Kv1.3 proteoliposomes) were recovered by centrifugation at 230,000 g. Kv1.3 proteoliposomes were stored at -80 °C until further use. Control liposomes were formed in the identical fashion as Kv1.3 proteoliposomes but do not contain any Kv1.3 protein. Both Kv1.3 proteoliposomes and control liposomes were examined by negative stain electron microscopy (EM) to assess their morphology and intactness using standard EM procedures [71]. Samples were deposited on continuous carbon coated copper mesh grids that were rendered hydrophilic by glow discharge using a Solarus Model 950 Advanced Plasma System (Gatan) in a 75/25 % Ar/O mixture and then stained with 2 % uranyl acetate. Micrographs were collected on a CM-120 (Phillips) operating at 120 keV at room temperature with a nominal pixel size of 2.33 angstroms per pixel (Å/pix) equipped with a Tem-Cam F224 slow scan CCD camera (Tietz).

### Recombinantly expressed Kv1.3 protein molecular weight characterization

To determine the oligomeric state of purified Kv1.3 proteins, Kv1.3 proteins solubilized in Fos-12 were exchanged into amphipols (A8-35) (Anatrace) following a modified protocol [72]. Briefly Kv1.3 proteins were incubated with a 5-fold excess of A8-35 (w/w) for 4 h at 4 °C. The mixture was then diluted to a 0.8-fold reduction of the Fos-12 critical micellular concentration (CMC) using Kv1.3 resuspension buffer and dialyzed overnight in a 100,000 MWCO slide-a-lyzer (Thermo) at 4 °C against detergent-free dialysis buffer in the presences of SM2 bio-beads (BioRad). Unbound A8-35 was removed by size exclusion chromatography using a 10/300 Superose 6 column (GE Healthcare). Kv1.3 proteins in Fos-12 and A8-35 were analyzed on a 4-16 % native gel (Invitrogen) according to manufacturer’s recommendations. Kv1.3 proteins in A8-35 were subjected to dephosphorylation using calf intestinal phosphatase (CIP) (New England Biolabs) according to manufacturer’s recommendations using a ratio of 1 CIP unit to 1 μg of Kv1.3 protein. Dephosphorylated proteins were analyzed on 8 % SDS-PAGE.

Negative stain EM was used to visualize purified Kv1.3 tetramers using standard EM procedures [71]. Samples were prepared in the same manner as mentioned above. Micrographs were collected using a Titan Krios EM (FEI) at 120 keV at room temperature equipped with a DE20 direct electron detector camera (Direct Electron) with a nominal pixel size of 1.26 Å/pix in an automated fashion using the Leginon software [73]. EM micrographs were uploaded to the Appion processing suite [74]. Kv1.3 tetramers were picked in a semi-automatic fashion using the template picker FindEM [75]. Two dimensional (2D) class averages were generated using the maximum likelihood alignment algorithm within the Xmipp package [76].

### Sec24a^341^ protein expression and purification

The Sec24a expression vector lacking the first 341 residues (Sec24a^341^) was a generous gift from Dr. Jonathan Goldberg (Howard Hughes Medical Institute, Memorial Sloan-Kettering Cancer Center) [39]. Sec24a^341^ proteins were expressed in the same manner as Kv1.3 proteins using the baculovirus expression system according to manufacturer’s recommendations.

Sec24a^341^ proteins were purified by resuspending infected Hi5 insect cells in Sec24a^341^ lysis buffer (25 mM Tris, 150 mM NaCl, 1 mM MgOAc, 5 mM 2-mercaptoethanol, one PIC tablet per 50 ml of buffer, 2 mL PopCulture reagent per 50 ml of buffer (EMD Millipore), pH 7.4) and incubated for 1 h at room temperature on a rotating carousel. Insoluble material was removed by centrifugation at 60,000 g. Cleared lysate supernatants were incubated with 3 ml of the HisPur™ Colbalt resin per 50 ml lysate supernatant and incubated at room temperature for 30 min. Loaded resin was collected by centrifugation at 2000 g and washed twice with Sec24a^341^ purification buffer A (25 mM Tris, 150 mM NaCl, 1 mM MgOAc, 5 mM 2-mercaptoethanol, pH 7.4). Sec24a^341^ proteins were eluted with Sec24a^341^ purification buffer B (purification buffer A with 500 mM imidazole). Eluents were analyzed on an 8 % SDS-PAGE gel and fractions containing Sec24a^341^ proteins were concentrated in a 30,000 kDa MWCO spin concentrator (Corning) to 10 mg/ml. Concentrated Sec24a^341^ proteins were loaded onto a FPLC Superose 6 10/300 GL size exclusion column (GE Healthcare) equilibrated with 2 column volumes of Sec24a^341^ purification buffer A using an ÄKTA FPLC. Sec24a^341^ proteins were analyzed on an 8 % SDS-PAGE gel for purity and fractions containing Sec24a^341^ were concentrated in a 30,000 kDa MWCO spin concentrator (Corning) to 1 mg/ml. Sec24a^341^ proteins were stored at -80 °C until further use.

### Kv1.3-Sec24a^341^ floatation assay

The Kv1.3-Sec24a *in vitro* association was detected using a modified membrane floatation assay. 15 μg of Sec24a^341^ protein was incubated with 15 μg of Kv1.3 proteoliposomes or 15 μg of Kv1.3 in detergent micelles in a total volume of 265 μl in floatation assay buffer (25 mM Tris, 150 mM NaCl, 160 mM KOAc, 1 mM MgOAc, pH 7.5) and 30 % sucrose w/v. Each reaction was incubated at 32 °C for 1 h. Reactions were first overlaid with 250 μl of floatation assay buffer containing 25 % sucrose w/v then followed by an additional overlay of 50 μl floatation assay buffer lacking sucrose. Each condition was centrifuged at 200,000 g for 1 h at 4 °C. Each layer was carefully removed and 50 μl samples were taken and mixed with 50 μl SDS-PAGE sample buffer. Control reactions were performed in parallel that contained Sec24a^341^, Sec24a^341^ in the presence of control liposomes, Kv1.3 micelles, Kv1.3 micelles in the presence of control liposomes, Sec24a^341^ and Kv1.3 micelles in the presence of control liposomes, and control liposomes alone. Samples were analyzed on an 8 % SDS-PAGE gel and were electro-transferred to nitrocellulose (Pall Life Sciences) at 4 °C in Towbin transfer buffer. Membranes were first blocked with 5 % non-fat dry milk w/v in TBST for 1 h at room temperature. Blocked membranes were incubated overnight at 4 °C with FSU120 and an anti-Sec24a primary antibody (Abcam). Host-specific secondary antibodies conjugated with an alkaline phosphatase reporter (Sigma) were added after three washes in TBST for 1 h at room temperature. Western blots were developed using the SIGMAFAST™ BCIP®/NBT tablets (Sigma). Antibodies were diluted in 5 % non-fat dry milk w/v in TBST to the following working dilutions: FSU120 (1:500), anti-Sec24 (1:250), secondary antibody (1:2500).

### Protein sequence alignments

All protein sequences were aligned using the Clustal Omega^©^ program as instructed [77]. Accession numbers are as follows: *Shaker;* CAA29917, mouse Kv1.1; NP_034725, mouse Kv1.2; NP_032443, mouse Kv1.3; AAI37668, mouse Kv1.4; NP_067250, mouse Kv1.5; AAG40241, human Sec24a; O95486.2, human Sec24b; O95487.2, human Sec24c; P53992.3, human Sec24d; O94855.2.

### Availability of supporting data

The data collected for the manuscript are presented within the article and Additional files 1, 2, 3, 4, 5, 6 and 7.

## Additional files

Additional file 1:
**Alignment of Kv channels shows conservation of the E443-E445-E447 acidic motif.** The C-terminus of *Shaker* and mouse Kv1.1 - Kv1.5 proteins were aligned from the selectivity filter (TVGYG; cyan box) onwards to show the conservation of the E443-E445-E447 residues (red box). A previously determined Kv1 channel ER export motif, VxxSL*,* (black box) shows little sequence homology across the Kv1.1 - Kv1.5 channels. A more recently determined Kv1.3 ER export motif was found, YMVIEE, (blue box) but it is not entirely conserved amongst the Kv1.1 - Kv1.5 channels. The green line indicates the membrane boundary of the S6 helix. Conserved residues are indicated (_*****_). Residues of similar charge are indicated (**:**). Residues of similar polarity are also indicated (**.**). Alignments were done using Clustal Omega^©^ sequence alignment program. Additional file 2:
**Basal biophysical properties of Kv1.3-eGFP channels.** (A) Schematic showing that the eGFP reporter (green barrel) was inserted directly after the start codon of Kv1.3 on the N-terminus. The Kv1.3-eGFP ∆C protein was truncated immediately after the S6 helix (dotted red line) at residue 427 of mouse Kv1.3. The three-glutamate residues of interest located at positions 443, 445, and 447 within the C-terminus of mouse Kv1.3 (red spheres) are indicated. (B) Representative current traces recorded from HEK 293 cells transiently transfected with cDNA encoding Kv1.3 alone (Kv1.3) or tagged with eGFP (Kv1.3-eGFP). Macroscopic currents were recorded from cell-attached patches held (V_h_) at -90mV and stepped in 10 mV increments to +40 mV (V_c_) using a 1000 millisecond pulse duration (P_d_) and a 60 s interpulse interval (T_s_). Mean current (± s.e.m.) is plotted for the family of generated voltage steps for five such recordings (right). Not significantly different by two-way ANOVA. (C) Same as in (B) but designed to resolve tail currents (5 mV increments; P_d_ = 50 millisecond, T_s_ = 10 s) that could be used to determine conductance and voltage-dependence (right). Normalized tail currents were fit with a Boltzmann relation to calculate voltage at half-activation (V_1/2_). Not significantly different V_1/2_, Student’s *t*-test. (D) Representative macroscopic currents normalized to visualize inactivation (left) and deactivation (middle) kinetics when patches were stimulated with a single step depolarization, V_h_ = -90 mV, V_c_ = +40 mV, P_d_ = 1000 millisecond. Bar graph (right) of the mean (± s.e.m.) peak current, inactivation time constant, and deactivation time constant for a population of recordings, n as specified. Not significantly different by Student’s *t*-test. Additional file 3:
**ER microsome isolation assay for determining the amount of Kv1.3-eGFP or mutant protein retained.** ER microsome isolation from HEK 293 cells. (A) Western blots probing for the ER luminal resident protein BiP, the Golgi luminal resident protein GCP60, the nuclear and perinuclear heat shock protein of 90 kDa (HSP90), and the cytosolic protein glyceraldehyde 3-phosphate dehydrogenase (GAPDH) during the microsome isolation centrifugation procedure. The homogenate, pre-clear supernatant (Preclear SN), and the pre-clear pellet contain BiP, GCP60, HSP90, and GAPDH. The ultracentrifugation supernatant (Ultra SN) is clear of BiP demonstrating that the ER microsomes remain intact through the centrifugation procedure. The Ultra SN contains GCP60 and GAPDH. The ultra pellet (Ultra P) has minor amounts of GCP60 and GAPDH but contains higher levels of BiP and HSP90, indicating that this fraction has been enriched for ER microsomes. (B) Western blots of the homogenate (top) and Ultra P (bottom) fractions from Kv1.3-eGFP and the respective mutants contain BiP, indicating that the ER microsomes from the cells expressing Kv1.3-eGFP or the respective mutant proteins remain intact through the isolation procedure. (C) Representative Western blots of whole cell lysates (top) and ER microsome enriched fractions (bottom) probed for Kv1.3-eGFP. An anti-actin probe is used to show equal protein loading across all lanes. Additional file 4:
**Knockdown of Sec24 in COS-1.** Individual Western blots probing for Sec24 proteins after treatment with the indicated siRNAs. An anti-actin probe is used to show equal protein loading across all lanes. Additional file 5:
**Kv1.3-eGFP trafficking in the presence of VSVG after siRNA mediated knockdown of Sec24.** Trafficking of Kv1.3-eGFP in the presences of the well-characterized vesicular stomatitis virus glycoprotein (VSVG) after siRNA mediated knockdown of the indicated Sec24 proteins. Confocal micrographs of Kv1.3-eGFP, VSVG-cerulean (pseudo colored red), or merged are indicated. Line scans of each fluorescent channel are shown. The white arrow in the merged image represents the placement and direction of the line scan. Scale bar = 5 μm. Additional file 6:
**Molecular weight characterization of recombinantly expressed Kv1.3 proteins.** Molecular weight determination of purified Kv1.3 proteins. (A) Size exclusion chromatogram of Kv1.3 proteins from two different membrane mimetic environments, n-dodecylphosphocholine (Fos-12, solid line) or amphipols (A8-35, dashed line). The shift in the A8-35 elution peak is due to an increased molecular mass associated with absorption of the A8-35 molecules. Profiles were normalized to the injection point (volume = 0) and the absorbance units (mAU) were auto-zeroed. (B) Native gel electrophoresis of eluted Kv1.3 proteins in either Fos-12 or A8-35. Kv1.3 proteins in A8-35 (right lane) show an elongated band around the 242kDa standard indicating that Kv1.3 in A8-35 yields tetrameric complexes. (C) Coomassie stained 8 % SDS-PAGE of Kv1.3 in A8-35 before and after the addition of calf intestinal phosphatase (CIP) (left and right middle lanes). After the addition of CIP the double band observed in the Kv1.3 control collapses to a single band. (D) Electron micrograph of negatively stained Kv1.3 tetramers in A8-35 (red circles). (E) Two dimensional (2D) reference free class averages of Kv1.3 tetramers in A8-35. The top view of Kv1.3 is shown in the middle right panel while the side views are shown in the top panels, middle left panel, and bottom panels. Additional file 7:
**Alignment of Sec24 shows conservation of the R750 and R752 residues.** Sec24 proteins were aligned to show that the R750 and R752 residues are conserved amongst all human Sec24 isoforms (red box). Conserved residues are indicated (_*_). Residues of identical charge are indicated (:). Residues of similar polarity are also indicated (.). Alignments were done using Clustal Omega^©^ sequence alignment program.

## Abbreviations

ANOVA: Analysis of variance
BSC40: Cercopithecus (African green) monkey kidney cell line - 40
COPII: Coat protein complex II
COS-1: African green monkey kidney fibroblast-like cell - 1
DAPI: 2-(4-amidinophenyl)-1H -indole-6-carboxamidine
DMEM: Dulbecco’s Modified Eagle Medium
eGFP: Enhanced green fluorescent protein
EM: Electron microscopy
ER: Endoplasmic reticulum
ERGIC: Endoplasmic reticulum Golgi intermediate compartment
FBS: Fetal bovine serum
HEK 293: Human embryonic kidney cell - 293
Kir: Inward rectifier potassium channel
Kv: Voltage-dependent potassium channel
MEM: Minimum essential media
PBS: Phosphate buffered saline
PMSF: Phenylmethanesulfonylfluoride
SDS-PAGE: Sodium dodecyl sulfate - polyacrylamide gel electrophoresis
TBS: Tris buffered saline
TBST: Tween-20 Tris-buffered saline
VSVG: Vesicular stomatitis virus glycoprotein
