# Supplementary material for: Kv1.3 contains an alternative C-terminal ER exit motif and is recruited into COPII vesicles by Sec24a
Source: BMC Biochem. 2015 Jul 10;16:16. doi: 10.1186/s12858-015-0045-6 (PMC4497498; doi:10.1186/s12858-015-0045-6)
Supplement: Additional file 1: — Alignment of Kv channels shows conservation of the E443-E445-E447 acidic motif. The C-terminus of Shaker and mouse Kv1.1 - Kv1.5 proteins were aligned from the selectivity filter (TVGYG; cyan box) onwards to show the conservation of the E443-E445-E447 residues (red box). A previously determined Kv1 channel ER export motif, VxxSL, (black box) shows little sequence homology across the Kv1.1 - Kv1.5 channels. A more recently determined Kv1.3 ER export motif was found, YMVIEE, (blue box) but it is not entirely conserved amongst the Kv1.1 - Kv1.5 channels. The green line indicates the membrane boundary of the S6 helix. Conserved residues are indicated (*). Residues of similar charge are indicated (:). Residues of similar polarity are also indicated (.). Alignments were done using Clustal Omega© sequence alignment program. [file 12858_2015_45_MOESM1_ESM.pdf]

Additional File 1

```
Shaker      --------------------tvgygdmtpvgvwgkivgslcaiagvltialpvpvivsnf   481
Kv1.1       --------------------tvgygdmypvtiggkivgslcaiagvltialpvpvivsnf   411
Kv1.2       --------------------tvgygdmvpttiggkivgslcaiagvltialpvpvivsnf   413
Kv1.3       --------------------tvgygdmhpvtiggkivgslcaiagvltialpvpvivsnf   436
Kv1.4       --------------------tvgygdmkpitvggkivgslcaiagvltialpvpvivsnf   564
Kv1.5       --------------------tvgygdmrpitvggkivgslcaiagvltialpvpvivsnf   508
                                ******* *  : ***************************

Shaker      nyfyhretdqeemqsqnfnhvtscpylpgtlgqhmkks--slsesssdmmdlddgvestp   539
Kv1.1       nyfyhretegeeq-aqllhv-ss-pnlasd-sdl-s-rrssstiskseymeieedmnnsi   465
Kv1.2       nyfyhretegeeq-aqylqv-tscpkipss-pdl-kksrsastisksdymeiqegvnnsn   469
Kv1.3       nyfyhretegeeq-aqymhv-gscqhlsssaeel-rkarsnstlskseymvieeggmnhs   493
Kv1.4       nyfyhreteneeq-tqltqnavscpylpsnllkkfrsstssslgdkseylemeegvkesl   623
Kv1.5       nyfyhretdheeq-aalkee-qggiqrresgldtg-g-qrk-vscskasfckt-ggplest   562
            ********: **  :   .   .     .               ..:.         .

Shaker      -gltethpgrsavapflgaqqqqqqpvas----slsmsidkqlqhplqhvtqtqlyqqq   594
Kv1.1       ahyrqanirt-gn-----ctt--adqncvnkskllltdv---------------------   495
Kv1.2       edfreenlkt-an-----ctl--antnyvnitkmltdv----------------------   499
Kv1.3       -afpqtpfkt-gn-statcttnnnpnscvnikkiftdv----------------------   528
Kv1.4       cgkeekcqgk-g------desetdknncsnakavetdv----------------------   654
Kv1.5       ds-----irr-gscplekchlkak--snvdlrrslyalcldtsre--------tdl----   602
                        .                  .       :
```
