# Supplementary figures and images for: Kv1.3 contains an alternative C-terminal ER exit motif and is recruited into COPII vesicles by Sec24a
Source: BMC Biochem. 2015 Jul 10;16:16. doi: 10.1186/s12858-015-0045-6 (PMC4497498; doi:10.1186/s12858-015-0045-6)

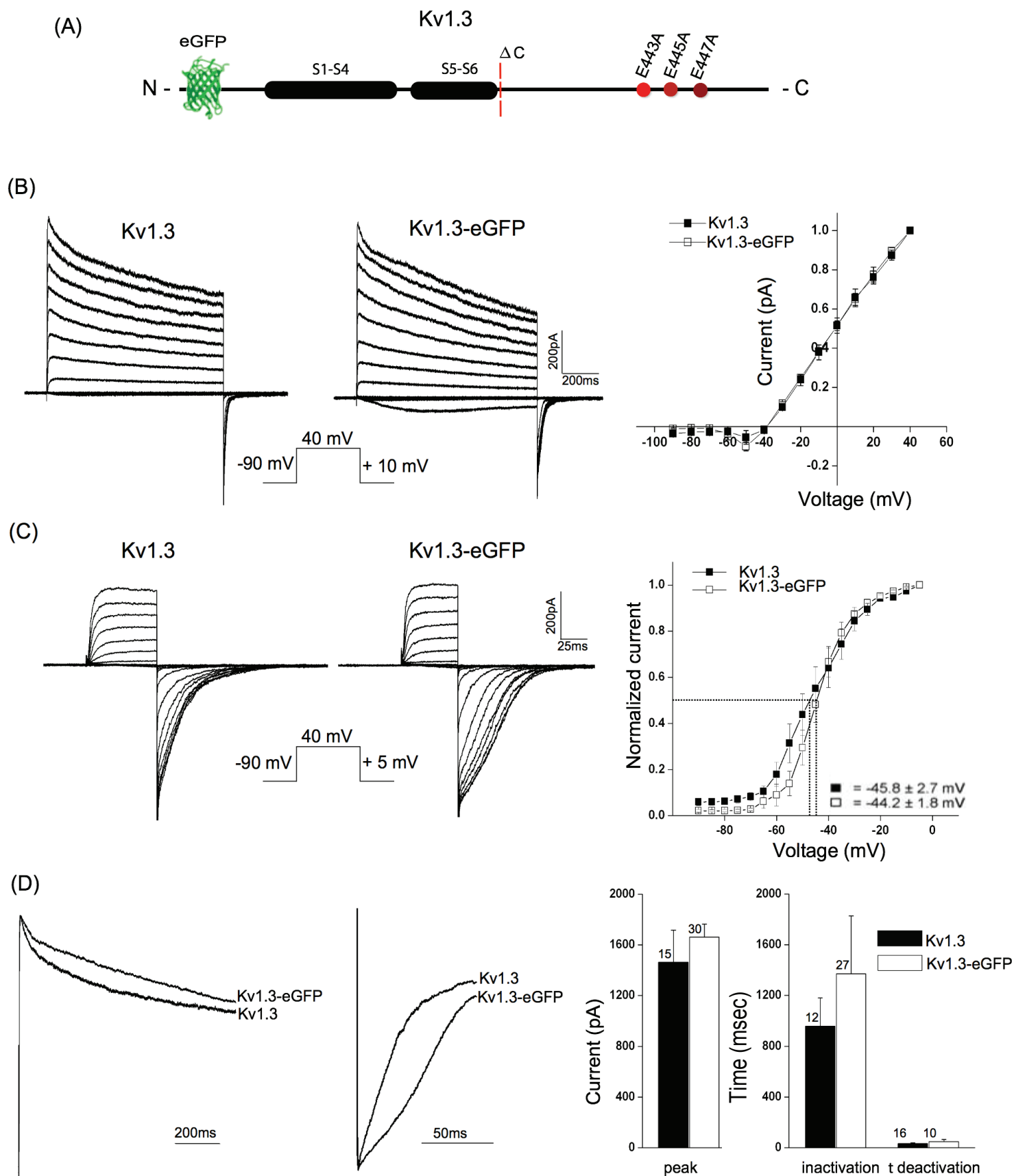

Supplement: Additional file 2: — Basal biophysical properties of Kv1.3-eGFP channels. (A) Schematic showing that the eGFP reporter (green barrel) was inserted directly after the start codon of Kv1.3 on the N-terminus. The Kv1.3-eGFP ∆C protein was truncated immediately after the S6 helix (dotted red line) at residue 427 of mouse Kv1.3. The three-glutamate residues of interest located at positions 443, 445, and 447 within the C-terminus of mouse Kv1.3 (red spheres) are indicated. (B) Representative current traces recorded from HEK 293 cells transiently transfected with cDNA encoding Kv1.3 alone (Kv1.3) or tagged with eGFP (Kv1.3-eGFP). Macroscopic currents were recorded from cell-attached patches held (Vh) at -90mV and stepped in 10 mV increments to +40 mV (Vc) using a 1000 millisecond pulse duration (Pd) and a 60 s interpulse interval (Ts). Mean current (± s.e.m.) is plotted for the family of generated voltage steps for five such recordings (right). Not significantly different by two-way ANOVA. (C) Same as in (B) but designed to resolve tail currents (5 mV increments; Pd = 50 millisecond, Ts = 10 s) that could be used to determine conductance and voltage-dependence (right). Normalized tail currents were fit with a Boltzmann relation to calculate voltage at half-activation (V1/2). Not significantly different V1/2, Student’s t-test. (D) Representative macroscopic currents normalized to visualize inactivation (left) and deactivation (middle) kinetics when patches were stimulated with a single step depolarization, Vh = -90 mV, Vc = +40 mV, Pd = 1000 millisecond. Bar graph (right) of the mean (± s.e.m.) peak current, inactivation time constant, and deactivation time constant for a population of recordings, n as specified. Not significantly different by Student’s t-test. [file 12858_2015_45_MOESM2_ESM.pdf]

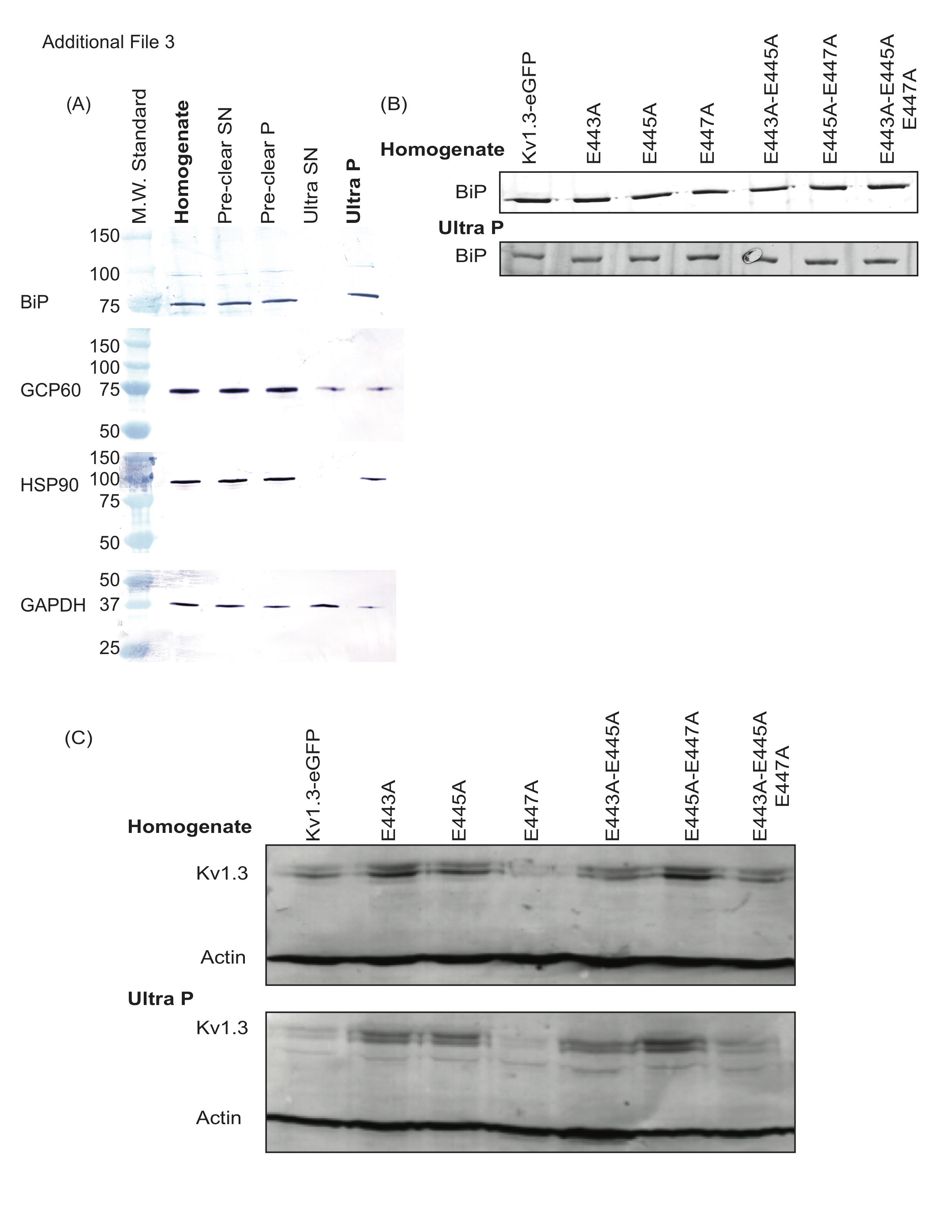

Supplement: Additional file 3: — ER microsome isolation assay for determining the amount of Kv1.3-eGFP or mutant protein retained. ER microsome isolation from HEK 293 cells. (A) Western blots probing for the ER luminal resident protein BiP, the Golgi luminal resident protein GCP60, the nuclear and perinuclear heat shock protein of 90 kDa (HSP90), and the cytosolic protein glyceraldehyde 3-phosphate dehydrogenase (GAPDH) during the microsome isolation centrifugation procedure. The homogenate, pre-clear supernatant (Preclear SN), and the pre-clear pellet contain BiP, GCP60, HSP90, and GAPDH. The ultracentrifugation supernatant (Ultra SN) is clear of BiP demonstrating that the ER microsomes remain intact through the centrifugation procedure. The Ultra SN contains GCP60 and GAPDH. The ultra pellet (Ultra P) has minor amounts of GCP60 and GAPDH but contains higher levels of BiP and HSP90, indicating that this fraction has been enriched for ER microsomes. (B) Western blots of the homogenate (top) and Ultra P (bottom) fractions from Kv1.3-eGFP and the respective mutants contain BiP, indicating that the ER microsomes from the cells expressing Kv1.3-eGFP or the respective mutant proteins remain intact through the isolation procedure. (C) Representative Western blots of whole cell lysates (top) and ER microsome enriched fractions (bottom) probed for Kv1.3-eGFP. An anti-actin probe is used to show equal protein loading across all lanes. [file 12858_2015_45_MOESM3_ESM.jpeg]

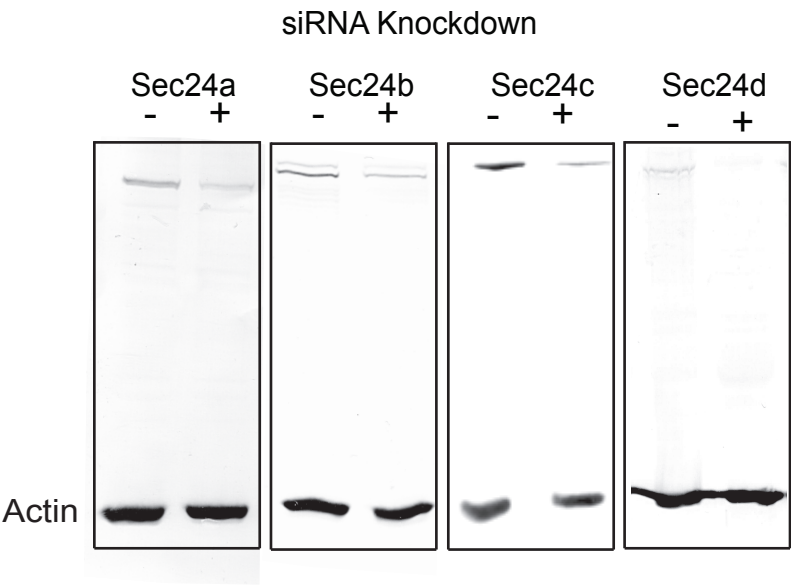

Supplement: Additional file 4: — Knockdown of Sec24 in COS-1. Individual Western blots probing for Sec24 proteins after treatment with the indicated siRNAs. An anti-actin probe is used to show equal protein loading across all lanes. [file 12858_2015_45_MOESM4_ESM.pdf]

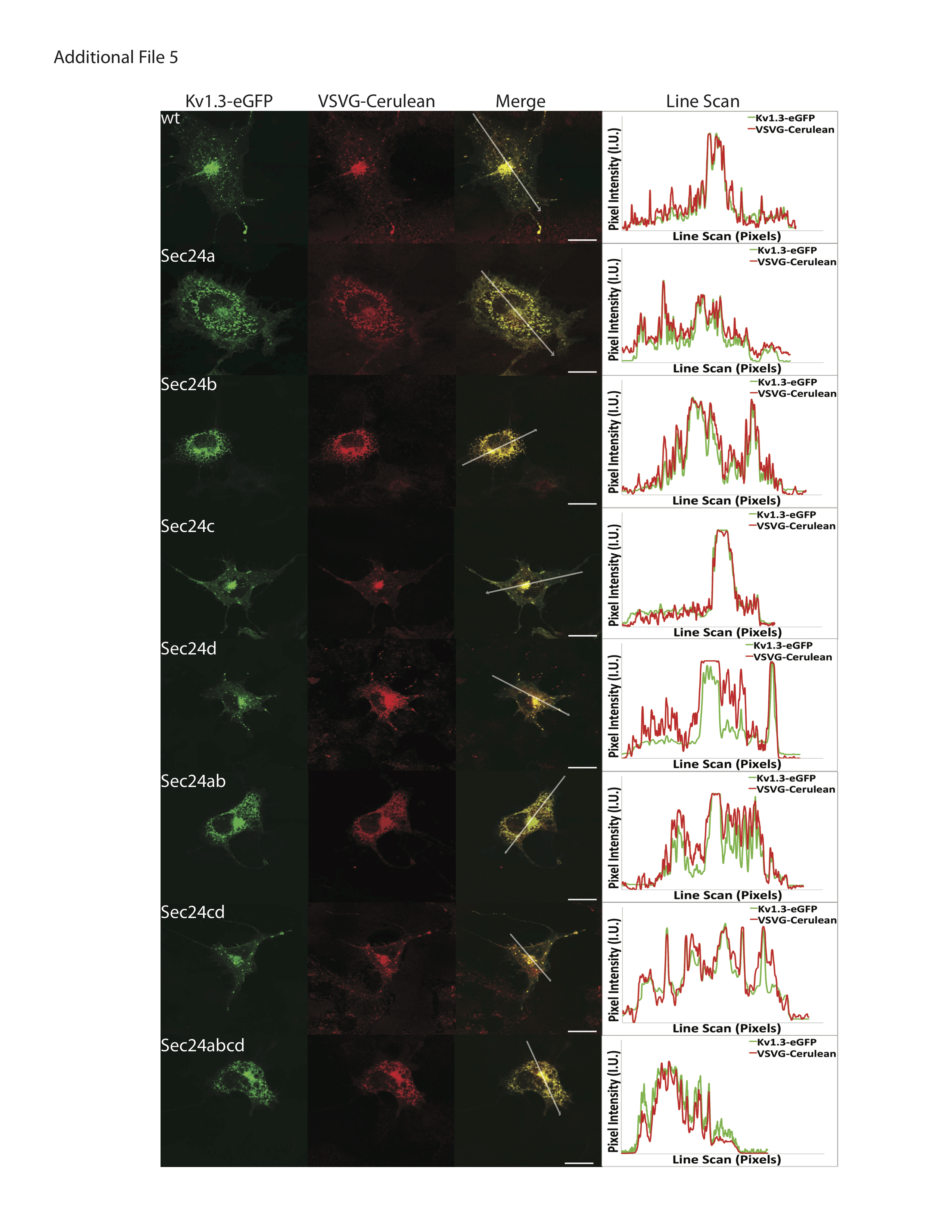

Supplement: Additional file 5: — Kv1.3-eGFP trafficking in the presence of VSVG after siRNA mediated knockdown of Sec24. Trafficking of Kv1.3-eGFP in the presences of the well-characterized vesicular stomatitis virus glycoprotein (VSVG) after siRNA mediated knockdown of the indicated Sec24 proteins. Confocal micrographs of Kv1.3-eGFP, VSVG-cerulean (pseudo colored red), or merged are indicated. Line scans of each fluorescent channel are shown. The white arrow in the merged image represents the placement and direction of the line scan. Scale bar = 5 μm. [file 12858_2015_45_MOESM5_ESM.jpeg]

Additional File 6

A)

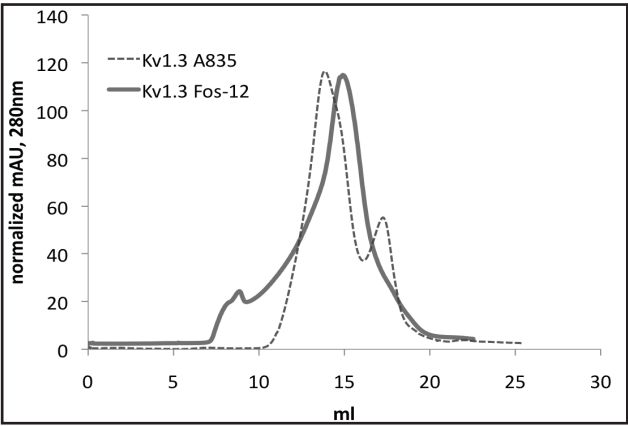

B)

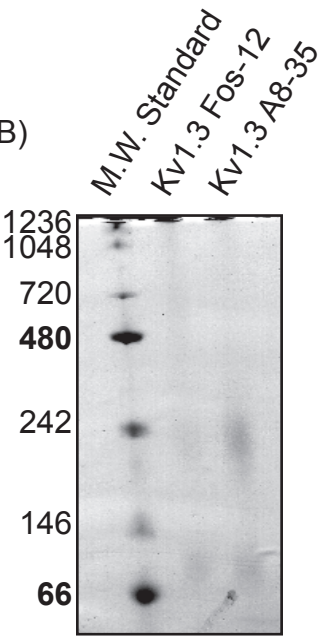

C)

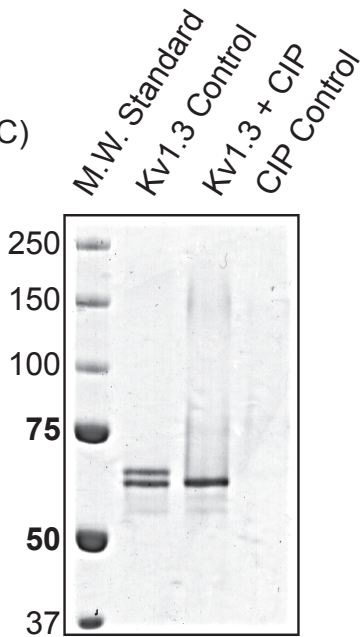

D)

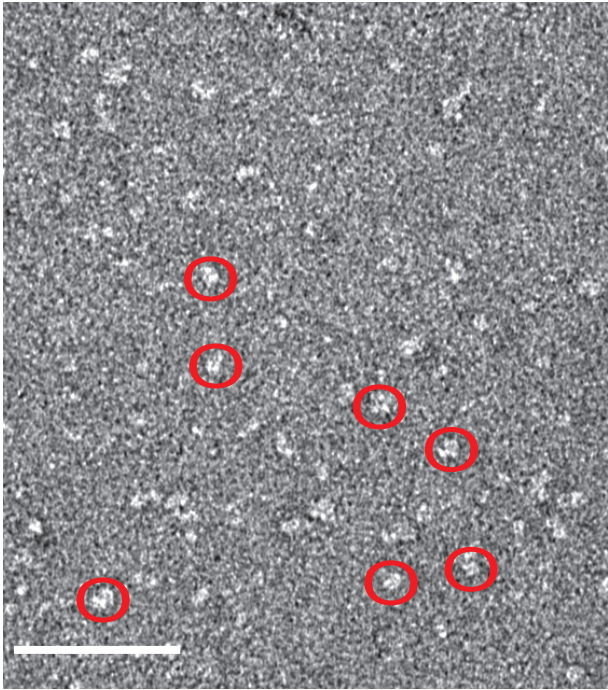

E)

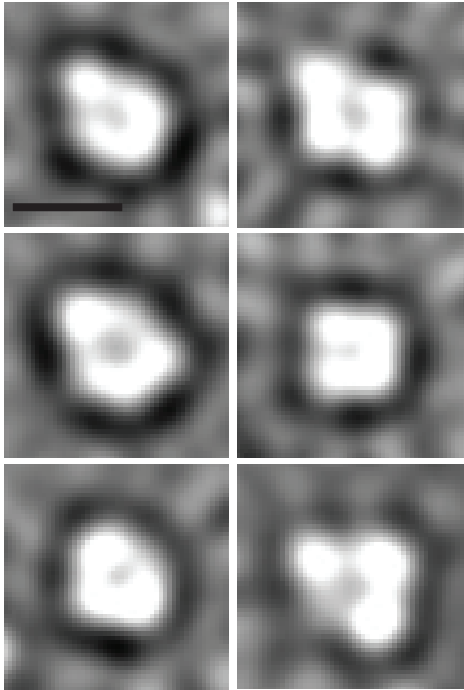

Supplement: Additional file 6: — Molecular weight characterization of recombinantly expressed Kv1.3 proteins. Molecular weight determination of purified Kv1.3 proteins. (A) Size exclusion chromatogram of Kv1.3 proteins from two different membrane mimetic environments, n-dodecylphosphocholine (Fos-12, solid line) or amphipols (A8-35, dashed line). The shift in the A8-35 elution peak is due to an increased molecular mass associated with absorption of the A8-35 molecules. Profiles were normalized to the injection point (volume = 0) and the absorbance units (mAU) were auto-zeroed. (B) Native gel electrophoresis of eluted Kv1.3 proteins in either Fos-12 or A8-35. Kv1.3 proteins in A8-35 (right lane) show an elongated band around the 242kDa standard indicating that Kv1.3 in A8-35 yields tetrameric complexes. (C) Coomassie stained 8 % SDS-PAGE of Kv1.3 in A8-35 before and after the addition of calf intestinal phosphatase (CIP) (left and right middle lanes). After the addition of CIP the double band observed in the Kv1.3 control collapses to a single band. (D) Electron micrograph of negatively stained Kv1.3 tetramers in A8-35 (red circles). (E) Two dimensional (2D) reference free class averages of Kv1.3 tetramers in A8-35. The top view of Kv1.3 is shown in the middle right panel while the side views are shown in the top panels, middle left panel, and bottom panels. [file 12858_2015_45_MOESM6_ESM.pdf]
