# Supplementary material for: Kv1.3 contains an alternative C-terminal ER exit motif and is recruited into COPII vesicles by Sec24a
Source: BMC Biochem. 2015 Jul 10;16:16. doi: 10.1186/s12858-015-0045-6 (PMC4497498; doi:10.1186/s12858-015-0045-6)
Supplement: Additional file 7: — Alignment of Sec24 shows conservation of the R750 and R752 residues. Sec24 proteins were aligned to show that the R750 and R752 residues are conserved amongst all human Sec24 isoforms (red box). Conserved residues are indicated (*). Residues of identical charge are indicated (:). Residues of similar polarity are also indicated (.). Alignments were done using Clustal Omega© sequence alignment program. [file 12858_2015_45_MOESM7_ESM.pdf]

Additional File 7

```
Sec24a SRYSAGSVYYYPSYHHQHNPVQVQKLQKELQRYLTRKIGFEAVMRIRCTKGLSIHTFHGN 765
Sec24b SKYSAGCIYYYPSFHYTHNPSQAEKLQKDLKRYLTRKIGFEAVMRIRCTKGLSMHTFHGN 940
Sec24c PQLTGGSVYKYASFQVENDQ---ERFLSDLRRDVQKVVGFDAVMRVRTSTGIRAVDFFGA 769
Sec24d PQLTGGTLYKYNNFQMHLDR---QQFLNDLRNDIEKKIGFDAIMRVRTSTGFRATDFFGG 707
       .: :.* :* * .::   :    ::: .:*:. : : :**:*:**:* :.*:    *.*
```
